# Challenges and Opportunities for Exploiting the Role of Zeolite Confinements for the Selective Hydrogenation of Acetylene

**DOI:** 10.1021/acsami.3c11935

**Published:** 2023-12-11

**Authors:** Jenna Vito, Manish Shetty

**Affiliations:** † Artie McFerrin Department of Chemical Engineering, Texas A&M University, 100 Spence Street, College Station, Texas 77843, United States

**Keywords:** zeolites, confined catalysis, shape selectivity, selective hydrogenations, single atom catalysts

## Abstract

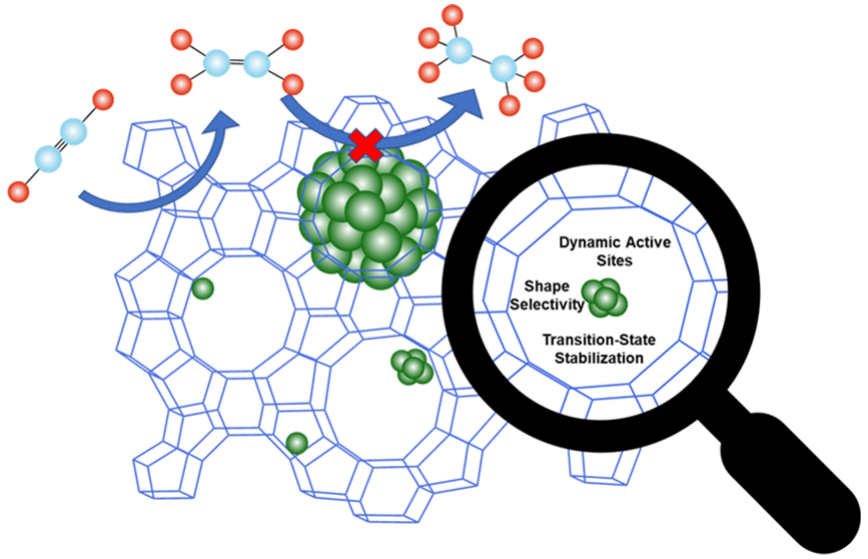

Zeolites, with their ordered crystalline porous structure, provide a unique opportunity to confine metal catalysts, whether single atoms (e.g., transition metal ions (TMIs)) or metal clusters, when used as a catalyst support. The confined environment has been shown to provide rate and selectivity enhancement across a variety of reactions via both steric and electronic effects, such as size exclusion and transition state stabilization. In this review, we provide a survey of various zeolite confined catalysts used for the semihydrogenation of acetylene highlighting their performance, defined by ethylene selectivity at full acetylene conversion, in relationship to the synthesis technique employed. Synthesis methods that ensure confinement with the catalyst transition metal location in the extra-framework positions are reported to have the highest selectivity to ethylene. However, the underlying molecular factors responsible for selective catalysis within confinement remain elusive due to the difficulty in deconvoluting individual effects. Through the careful use of a combination of characterization and spectroscopic methods, insights into the relationship between the properties of zeolite confined catalysts and their performance have been explored in other works for a variety of reactions. More specifically, *operando* spectroscopy studies have revealed the dynamic behavior of zeolite confined catalysts under various conditions implying that the structure and properties observed *ex situ* do not always match those of the active catalyst under reaction conditions. Applying this type of analysis to acetylene semihydrogenation, a simple gas phase reaction, can help elucidate the structure–function relationship of zeolite confined catalysts allowing for more informed design choices and consequently their application to a wider variety of more complex reactions such as the liquid phase hydrogenation of alkynols where solvent effects must also be considered in addition to those of confinement.

## 1 Introduction

Acetylene semihydrogenation is an industrially significant reaction as it is required to produce high purity ethylene for polyethylene production. Ethylene is typically sourced from naphtha cracking, which results in anywhere from 0.5 to 8 wt % acetylene in the product stream.^1^ The Ziegler–Natta polymerization catalyst is poisoned by acetylene, thereby, requiring concentrations of acetylene in the feed stream to be reduced to low ppm levels (<5 ppm).^2−4^ The removal of acetylene by typical separation methods (i.e., distillation or size-based separations) is difficult and energy-intensive, making them undesirable. Hence, hydrogenation has been adopted to remove the acetylene impurity.^5−7^ Specifically, the semihydrogenation of acetylene, in which the acetylene molecule undergoes only one hydrogenation to ethylene instead of fully hydrogenating to ethane, is desirable to maximize the amount of ethylene and for the efficient use of H_2_. Industrially, this reaction takes place under either front-end conditions, in which the feed includes excess H_2_ and C_1_ impurities such as methane and carbon monoxide, or tail-end condition, where C_1_ molecules have been removed, and H_2_ is added in stoichiometric amounts.^8,9^ Both of these feeds utilize Pd based catalysts, moderate temperatures (∼313–393 K), and pressures between 15 and 35 bar.^8^ Laboratory scale reactions typically take place within the same temperature range, but at ambient pressure and often with a pure acetylene feed stream for more direct evaluation of catalysts’ ability to hydrogenate acetylene.^8,10,11^ More detailed information about the reaction conditions for each of the laboratory scale acetylene hydrogenation reactions referenced throughout this work can be found in Table S1.

Common hydrogenation metals, including Pd, Pt, Ni, Rh, Co, Fe, and Cu, on a variety of supports (e.g., silica (SiO_2_), alumina (Al_2_O_3_), and zeolites) have been implemented in the design of catalysts for acetylene hydrogenation.^1,12^ The industrial standard for hydrogenation reactions has historically been the Lindlar catalyst, a palladium (Pd) catalyst on a carbonate support that is selectively poisoned with both lead (Pb) and quinoline. While this catalyst is effective at hydrogenating acetylene to ethylene, it also readily hydrogenates ethylene to ethane resulting in less than optimal use of H_2_ along with loss of ethylene for polymerization.^1,13^ A more recent commercial alternative is a bimetallic Pd–Ag catalyst on a γ-alumina (γ-Al_2_O_3_) support which features low metal loadings (0.03–0.04 wt % Pd) and good ethylene selectivity (∼60–90%).^12,14,15^ While these two catalysts, among others, such as Pd/TiO_2_,^16,17^ Ni/SiO_2_,^18,19^ and Pd or Ni intermetallic compounds^20−22^ support high acetylene conversion giving the required ethylene purity for downstream processes, they still leave something to be desired in terms of ethylene selectivity. For instance, unmodified Pd/TiO_2_ catalysts were reported to have an ethylene loss, defined as a negative value for the change in ethylene concentration between reactor inlet and outlet divided by the initial ethylene concentration, of 81%.^16,17^ While some catalysts have shown ethylene selectivity >90%, they make a compromise on activity and catalyst lifetime leading to a need for further study to develop active and selective catalysts.^1^

In addition to overhydrogenation of acetylene to ethane, the selectivity to ethylene can be further reduced by the formation of green oils, which are liquid C_4+_ olefins and paraffins formed from the oligomerization of acetylene and/or ethylene.^23,24^ Most hydrogenation catalysts, including Pd and Ni, also support the oligomerization reaction making the design of selective catalysts a challenge.^25^ One technique that has been successful in reducing green oil formation is the modification of the active catalysts with another inert metal to form an intermetallic or alloy.^21,26^ Dilution with inert metals alter the activity and selectivity of the catalysts often through changes to adsorption energies and configurations of reactants and key intermediates.^14,21,26,29^ Furthermore, if the ratio of active to inert metal is low, this can result in site isolation of the active metal, thus decreasing the occurrence of polymerization reactions that require two or more neighboring active sites.^15,27^ For instance, Ni_*x*_Ga/SiO_2_ intermetallic catalysts were shown to have a decreased selectivity to C_4+_ products compared to Ni/SiO_2_ (∼17% compared to 40%) as the formation of the intermetallic phase increased the isolation and electron density of Ni resulting in weaker ethylene adsorption, making it unfavorable for the polymerization reaction.^25^ This is in contrast to the findings of Yang et al., in their study of zeolite confined Rh atoms and clusters for ethylene hydrogenation and dimerization, where in they find that single Rh species exhibit a higher selectivity for ethylene dimerization than clustered Rh particles (81.5 wt % C_4_ molecules in the product stream versus 35.9 wt %).^28^ This contradiction shows the need for careful analysis of these materials as well as more absolutely defining the impact of the catalyst structure on reaction trajectories.

Along with decreasing the amount of C_4+_ products, having weakly adsorbed ethylene is key to preventing over hydrogenation. If the desorption energy of ethylene is less than the energy barrier for its hydrogenation to ethane, then ethylene will desorb rather than react further to ethane. This scenario especially exists when ethylene is adsorbed to single metal atom catalysts.^19,21^ Single atom catalysts (SACs) can be isolated in metal alloys or dispersed on various supports, such as SiO_2_, Al_2_O_3_, metal organic frameworks (MOFs), or zeolites, with low loading of the active metal species.^1,30^ Pei et al.^31^ synthesized several variations Ag alloyed Pd catalysts on silica supports to selectively hydrogenate acetylene. They found the catalysts showed enhanced ethylene selectivity (>90% compared to ∼0% for a 1 wt % PdAg/Al_2_O_3_ control) while maintaining acetylene conversion above 90% despite having very low Pd loadings (ppm level). Similar to isolation via alloying, Ma et al.,^22^ isolated Ni atoms within a high entropy intermetallic (HEI) via the multimetallization of NiGa. They deposited HEI nanoparticles on a SiO_2_ support (HEI/SiO_2_) and probed its performance for acetylene semihydrogenation. The HEI/SiO_2_ catalyst was able to achieve >90% selectivity at full acetylene conversion and did not support the formation of any green oils whereas the selectivity of the NiGa/SiO_2_ control dropped to −60% once reaching full conversion.^22^ While SACs have demonstrated excellent selectivity to ethylene, they appear to be plagued by lower activity such that they require higher temperatures to reach the same conversion as the control catalysts referenced in the above experiments. However, many times reaction rates and conversions are not normalized to the amount of metal loading in the reactor (i.e., reporting rates per gram), so interpreting activity based on the reaction temperature alone can be misleading.

An alternative to SACs that also exploits the difference in adsorption energy of acetylene and ethylene is to utilize a surface layer that selectively prevents the adsorption of ethylene. For instance, Choi and co-workers have explored the use of polymer supports for the selective hydrogenation of acetylene using Pd catalysts.^32,33^ They observed that various polymers, such as polyphenylene sulfide (PPS), could interact with the metal catalyst resulting in a polymer coating around the Pd nanoparticles that prevented the adsorption and activation of hydrogen.^33^ However, since acetylene is strongly attracted to the Pd surface it is able to “pull away” the polymer coating allowing for its adsorption and activation of hydrogen through the substrate assisted mechanism.^33^ Since ethylene binds more weakly to the surface, it is unable to adsorb and activate hydrogen through the same mechanism, thereby preventing its undesirable hydrogenation to ethane. Through this mechanism, the Pd/PPS catalyst was able to achieve a much higher ethylene selectivity (∼72%) than the traditional Pd/SiO_2_ catalyst (∼48%) under the same conditions. Additionally, the “strength” of the polymer coating can be tailored by altering the ligating groups on the end of polymer chain that interact with the metal surface.^32^ This implies that polymer/ligating agent selection can be chosen in order to exploit adsorption energy differences between other molecules for any series reaction where the intermediate produced is desirable.

A promising area of exploration to address the activity and selectivity issues highlighted above for SACs is the development of catalysts utilizing zeolite supports. Zeolites, which are crystalline aluminosilicates, have been used for shape-selective catalysis. They offer a unique opportunity to enhance selectivity based on size differences between desired and undesired products.^34^ Moreover, the zeolite confinements have been shown to stabilize transition-states for a number of reactions, including selective hydrogenations of alkenes and alkynes, selective catalytic reduction (SCR) of nitrous oxides (NO_*x*_), and selective isomerization reactions, which can result in selectivity enhancement.^35−37^ For instance, in the isomerization of d-glucose, it was found that titanium (Ti) catalysts within the confinement of small pore zeolites preferentially stabilized transition states leading to the formation of the sorbose product over the transition states that result in the competing fructose product.^38^ This resulted in the sorbose/fructose ratio to increase from <1 to >10 when the zeolite window diameter decreased from 0.7 nm (Beta) to 0.55 nm (MFI).^38^ Additionally, the local electric field caused by zeolite confinement has been suggested to promote increases in the reaction rates. A key step in hydrogenation reactions is the activation of dihydrogen molecules, which has been proposed to be promoted by the local electric field in zeolite confinements. Specifically, propene hydrogenation has seen rates five times greater within the confined environment of zeolite MCM-22 (pore window 4.1 × 5.1 Å) compared to unconfined catalyst on the zeolite surface.^39^ Overall, zeolite confined catalysts have been shown to enhance selectivity and reactivity for a number of reactions through steric and promotion pathways (i.e., electric fields) that affect stabilization of transition states and shape selectivity.

Using zeolites as hosts for SACs has the potential for a synergistic relationship that results in significant selectivity and rate enhancement for the selective hydrogenation of acetylene. Some of the smallest pore zeolites, such as chabazite (CHA), have a pore size (3.8 × 3.8 Å) smaller than the kinetic diameter of ethane (4.4 Å) thereby theoretically preventing the formation of undesired products, whether ethane or the even larger oligomers.^6,34^ In the case of larger pore zeolites, pairing with SACs, can still result in selectivity enhancement on the basis of the ethylene desorption energy being less than the barrier for further hydrogenation. Additionally, single atom sites will prevent oligomerization when the mechanism requires neighboring metal atoms, and they appear to reduce the formation of metal hydrides which are required for full hydrogenation.^15,27^ While it has been noted that SACs can suffer from decreased activity and stability compared to nanoparticle catalysts, utilizing zeolite supports may overcome these shortcomings with their ability to enhance rates and stabilize the catalytic species.^1,20,35^ Additional stabilization of catalytic species within confinement can be provided through the use of ligands which bind to the support and prevent sintering.^40,41^ With so many avenues toward rate and selectivity enhancement, it is difficult to probe individual effects leading many to broadly attribute their findings to a generic combination of steric and electronic effects. The situation becomes even more complex when venturing into the realm of liquid-phase reactions where the additional interactions between solvent molecules, reacting substrate, and active sites must be considered in addition to any stabilization provided by zeolite confinements.^42−44^ As such, it would be beneficial to first determine the impact of confinement for simple gas phase reactions (e.g., acetylene hydrogenation) prior to moving to more complex molecules and liquid phase reactions.

In this review, zeolite confined catalysts used for the semihydrogenation of acetylene are highlighted with a specific emphasis on the synthesis and characterization of confined catalysts and the need to elucidate the relationships between substrate and active site interactions, coordination environment, and transition state stabilization to inform better zeolite confined catalyst design. We first highlight the reaction mechanism of acetylene hydrogenation including elucidation of the subtleties that occur in the confined zeolite environment. Next, we cover the synthetic and postsynthetic techniques used for the synthesis of the confined catalysts including zeolite-confined SACs and clusters. Then, we focus on the suite of characterization tools including *operando* spectroscopy to probe the physical and electronic structure and restructuring of the active sites. Finally, we focus on the utility of the confined SACs on liquid–solid interfaces for the condensed phase hydrogenation of alkynols to alkenols, along with a brief overview of additional supports that provide confinement.

Overall, this review provides a survey of zeolite confined catalysts applied to the selective semihydrogenation of acetylene within the context of identifying molecular factors responsible for observed selectivity enhancement compared to catalysts not within confined environments. This unique perspective was taken with the intent of encouraging the community to further explore the impact of zeolite confinements, specifically through the careful execution of characterization techniques, including *operando* spectroscopy, to allow for making more informed decisions regarding zeolite confined catalyst design and their application to a wider variety of reactions.

## 2 Reaction Mechanism and Potential Energy Surface of Zeolite Confined Catalysts

**Figure 1:**
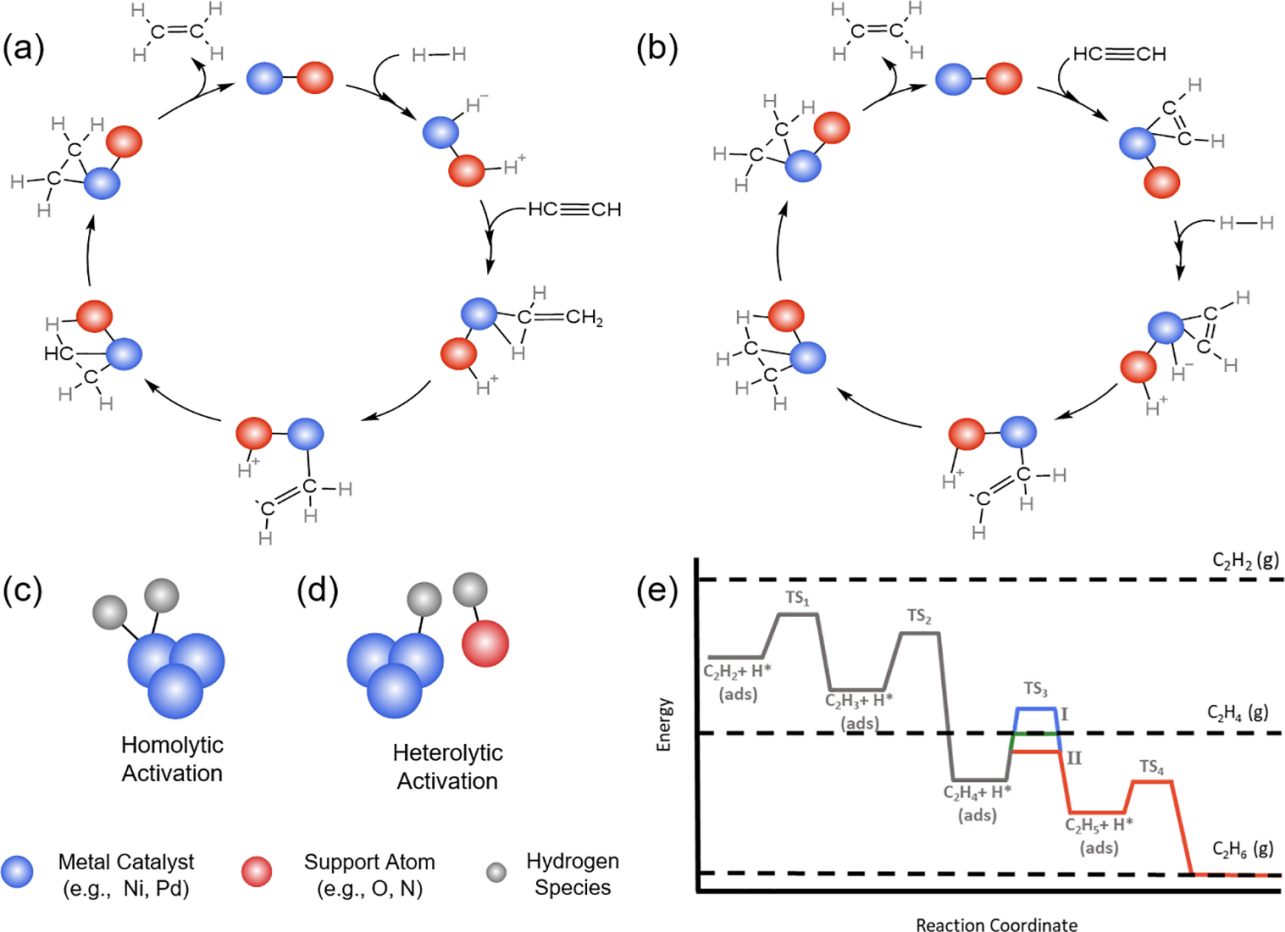
Possible elementary steps for acetylene hydrogenation to ethylene in zeolite confined catalysts: (a) Horiuti-Polanyi mechanism and (b) substrate assisted mechanism. The Horiuti-Polanyi mechanism involves the independent adsorption and activation H_2_ and the substrate. H_2_ activation is depicted first here, followed by substrate adsorption and subsequent hydrogenation. The substrate assisted mechanism occurs when the metal cannot directly activate H_2_; therefore the reactant molecule must adsorb first to assist with H_2_ activation. (c) Depiction of homolytic H_2_ activation over a metal catalyst resulting in two uncharged surface hydrogen species. (d) Depiction of heterolytic H_2_ activation over a metal catalyst and support atom resulting in a hydride and a proton. (e) Potential energy landscape depicting acetylene hydrogenation to ethylene and ethane. If the energy for ethylene desorption (middle dashed line) is less than the barrier for hydrogenation to ethane (blue line), ethylene will be selectively produced. However, if this energy barrier is lower than desorption, then ethylene will be hydrogenated to ethane (red pathway). Reproduced/adapted with permission from ref (1). Copyright 2022 Elsevier.

Depending on the catalyst utilized, acetylene hydrogenation has been seen to follow two different mechanisms–the Horiuti-Polanyi mechanism or a substrate assisted (associative) mechanism.^1,12,37,45^ Traditionally, acetylene hydrogenation over heterogeneous catalysts follows the Horiuti-Polanyi mechanism, detailed in Figure 1a.^1^ In brief, this mechanism involves the independent adsorption of both dihydrogen and acetylene. Upon its adsorption, dihydrogen is activated by the catalyst either heterolytically (*vide* infra) by splitting into a hydride and proton (as seen in Figure 1a), or homolytically, which forms an uncharged metal hydride or surface hydrogen species. This activation is followed by hydrogen addition to the adsorbed acetylene substrate. Alternatively, the reaction can follow the substrate-assisted pathway, in which the first step in the mechanism is the adsorption of the substrate molecule, when the metal catalyst alone cannot activate the dihydrogen molecules (Figure 1b).^1,12^ The adsorbed substrate then assists in the activation of the dihydrogen species through electron transfer to the metal catalyst.^1^

**Figure 2:**
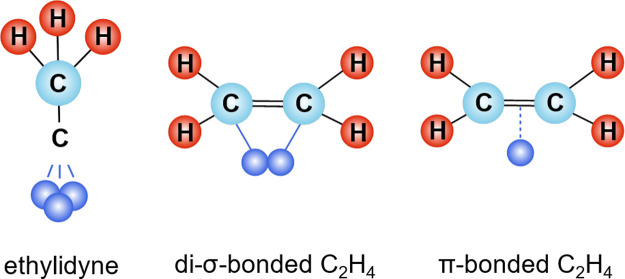
Configurations of ethylene adsorption on different metal catalysts. Reproduced/adapted with permission from ref (1). Copyright 2022 Elsevier.

Regardless of the overall mechanism, dihydrogen activation is a critical step in the hydrogenation reactions. Activation can be either homolytic (Figure 1c), where the dihydrogen bond is cleaved by the metal catalyst and the resulting two hydrogen species remain adsorbed to the metal, or heterolytically (Figure 1d), where the dihydrogen splits into a hydride and proton adsorbed onto the metal catalyst and a neighboring more basic atom, respectively.^1,46^ Catalysts supported on materials that contain proton accepting atoms, such as nitrogen and oxygen, SACs and metal oxides can all activate dihydrogen heterolytically.^1,46^ This ability to activate dihydrogen is what makes Pd a good hydrogenation catalyst, however Pd clusters activate via homolytic cleavage which results in the formation of metal hydrides—a key factor in over hydrogenation.^1,15,27,47^ In addition to the preference for heterolytic activation, SACs can enhance selectivity to ethylene based on the mode of ethylene adsorption. SACs stabilize the π-bonding of ethylene with the catalyst surface compared to di-σ-bonding or ethylidyne on metal aggregates (Figure 2).^1^ The desorption enthalpy for the π-bonded ethylene is lower than the barrier for further hydrogenation to ethane theoretically allowing for an inherent selectivity to ethylene (Figure 1e).^48,49^

In zeolite confinements, SACs are coordinated with framework oxygen atoms, which can act as the proton acceptor in the heterolytic dissociation of H_2_. Additionally, it has been suggested that confinement offers a local electric field that can make dihydrogen cleavage easier, making them a good candidate for selection as catalyst supports to be applied to hydrogenations (*vide infra*).^37^ Interestingly, large (FAU, pore window 7.4 × 7.4 Å) and small (CHA, pore window 3.8 × 3.8 Å) pore zeolites containing Ni SACs have been suggested to follow different mechanisms for acetylene hydrogenation.^45,50^ It was suggested that the smaller pore zeolite follows the Horiuti-Polanyi mechanism, while the larger pore follows the associative mechanism. The local electric field within the small pore was believed to be stronger thereby allowing for the direct activation of the dihydrogen molecule (*vide infra*). Regardless of mechanism, both catalysts were observed to be highly selective (>90%) to ethylene indicating that full hydrogenation of acetylene to ethane was unfavorable.^37,45,50^ However, when testing the ability to hydrogenate ethylene alone, significant conversion was still seen, implying that the barrier for further hydrogenation is not higher than that of ethylene desorption as would be expected for SACs.

## 3 Synthesis Techniques and Their Impact on Catalyst Performance

**Figure 3:**
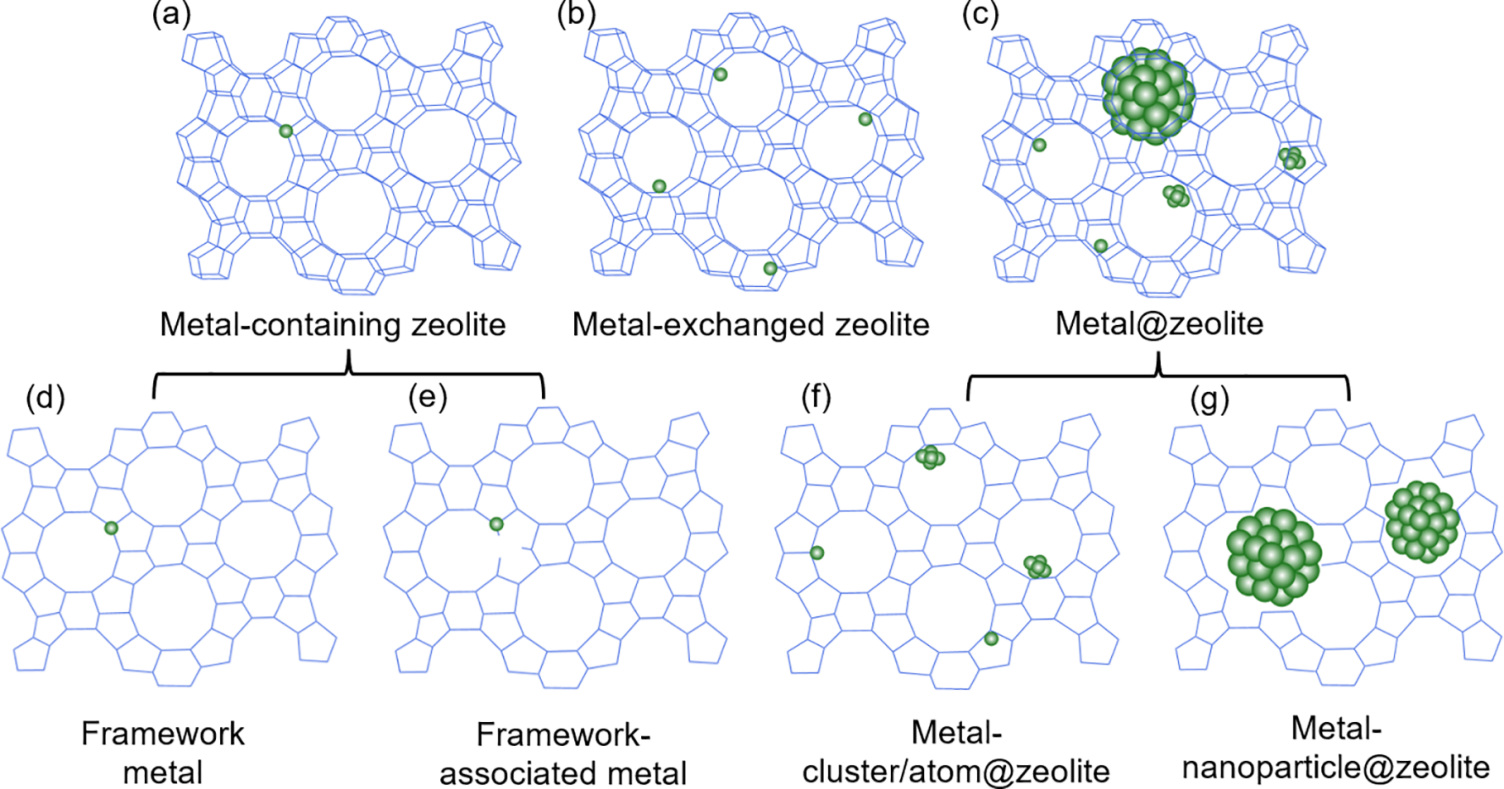
Locations for transition metal atoms/ions/clusters on zeolites. (a) Metal containing zeolites with (d) framework metal sites (no M–OH bonds) or (e) framework-associated sites (both M–O-Si and M–OH bonds); (b) metal exchanged zeolites; (c) metal@zeolites with (f) metal-clusters or atoms in the extra-framework sites or (g) metal nanoparticles >1 nm that disrupt the zeolite structure. Reproduced/adapted with permission from ref (36). Copyright 2022 American Chemical Society.

When utilizing zeolites as a catalyst support, it is crucial to consider the location of the catalyst on the support as it has a direct impact on the properties and thereby the functionality of the catalyst. More specifically, zeolites have framework (Figure 3a) and extra-framework (Figure 3b) locations which can host the active metal species to form SACs and clusters.^36^ Transition metals can be substituted into framework and framework associated sites in place of the aluminum (Al) atoms following a post synthetic dealumination process (Figure 3d, e). These metal atoms are left in an electron deficient state through charge transfer to Si through the Si–O–M (metal) bonds which allows the metal to accept electrons and function as a Lewis-acid catalyst.^36,37^ Framework incorporated metal–zeolite catalysts of this type have been used to catalyze reactions including various biomass transformations and olefin epoxidations.^51−53^ In the case of hydrogenation reactions, the extra-framework sites are utilized to host transition metal ions (TMIs) or clusters (Figure 3b, c).

Single TMIs are typically in their cationic form and are used to balance the negative charge of [AlO_4_]^−^ units in the zeolite framework in place of protons or alkali metal ions. Generally, bivalent cations, such as Pd^2+^ and Ni^2+^, are most stable when coordinated with four framework oxygen atoms and balanced by two framework Al sites.^37,54^ When located in these positions, the ions are thermodynamically stable such that they cannot be displaced by additional ion exchange with alkali metal ions.^37^ However, the paired Al site requirement imposes a limitation on catalyst loading since the factors that control Al location in the zeolite framework are not well-defined.^55−57^ A variety of synthesis methods have been developed and employed to introduce transition metal species into extra-framework sites including both postsynthetic and hydrothermal methods.^36^ In this section, we review several examples of zeolite confined catalysts synthesized for the semihydrogenation of acetylene. We cover post-synthetic impregnation and ion-exchange followed by direct hydrothermal synthesis techniques while highlighting catalyst performance and its relationship to the synthesis technique employed.

### 3.1 Post Synthesis Techniques

Ion exchange and incipient wetness synthesis methods are by far the simplest methods to introduce catalysts into zeolite confinement. The zeolite framework is negatively charged. Hence, the zeolites’ channels contain freely moving cations (Na^+^, K^+^, NH_4_^+^, H^+^) to maintain a neutral charge. Both techniques involve making an aqueous solution with a metal salt precursor (e.g., metal nitrates) to replace the pre-existing cations with the desired metal cations from the precursor solution. Impregnation techniques utilize a volume of the metal ion solution that is less than the total pore volume of support while ion exchange methods use a larger volume of solution such that both catalyst and support are dispersed in the solution.^58^ While these synthesis methods are relatively straightforward, they have shown a lack of uniformity in dispersion of the extra-framework metal sites across the zeolite support and a number of metal sites being present on the external surface of the zeolite, as opposed to within the channels. This is evident in the study by Corbin et al., where exchanging Ni ions with H-form zeolite A (pore window 4.1 × 4.1 Å) was used to prohibit the hydrogenation of butadiene in a mixed stream of butadiene (kinetic diameter 4.2 Å) and acetylene (kinetic diameter 3.3 Å).^6^ Initially, no reduction in butadiene hydrogenation was seen when using the zeolite support compared to a commercially available alumina (Al_2_O_3_) supported Ni catalyst.^6^ Upon including thiophene or phosphene, which are bulky catalyst poisons containing sulfur and phosphorus, respectively, butadiene hydrogenation decreased from 100% to around 30%. Due to the large size of the poisons, it was suggested that they cannot diffuse into the pores of the zeolite support, so they will exclusively poison Ni sites on the external surface of zeolites (i.e., those not within confinements). Thus, indicating that the hydrogenation of butadiene was primarily catalyzed by the external Ni sites and that the zeolite confinement can enhance product selectivity via size exclusion. Interestingly, in addition to the reduction in butadiene hydrogenation, this work reported an ethylene selectivity enhancement for their Ni/zeolite A catalysts compared to commercially available alumina (Al_2_O_3_) supported Ni catalysts but did not delve into what differences between the supports (e.g., confinement) may be responsible for this observation. While this shows the validity of using zeolite supports for size-based selectivity enhancement, it also emphasizes the importance of ensuring catalytic species are located in the extra-framework sites to achieve size exclusion-based selectivity.

Similar studies with Pd exchanged zeolites were also able to achieve similar activity and selectivity compared to a standard commercial catalyst (0.04 wt % Pd/Al_2_O_3_) without selective poisoning.^5,59^ Denkewicz et al. noted having greater metal site dispersion over the support decreased oligomer formation due to a lack of adjacent sites, and that a higher concentration of acid sites increased oligomer selectivity.^5^ The acidity, or the Si/Al ratio, has a direct impact on metal dispersion, as the number of exchangeable cations is directly proportional to the number of Al sites in the framework. Higher Si/Al ratios mean there are fewer exchangeable cations and therefore, in the case of single atom catalysts, more dispersion of metal sites on the zeolite resulting in a decrease in oligomerizations, which require adjacent transition metal sites. For the same metal loading across supports, higher Si/Al would have the opposite effect, as it could lead to the formation of larger metal agglomerates and consequently lower dispersion.

In addition to being metal-catalyzed, oligomerization is also supported by the Brønsted acid sites (the charge balancing protons) of zeolites. Hence, the reduction in the number of these sites can increase selectivity.^60,61^ Specifically, in the Denkewicz et al. study, the exchange of charge balancing protons with Na^+^ ions was found to decrease oligomerization (selectivity to oligomers decreasing from 25% pre-exchange to 20% postexchange) suggesting that support acidity also plays a significant role in determining product selectivity.^5^ Overall, it can be seen that not only is the location of TMIs crucial for catalyst performance but also the acidity of the zeolite support. In the case of zeolites, less acidic (high Si/Al ratio) supports will prevent acid catalyzed oligomerization but at the expense of lower metal loadings (SACs) or decreased dispersion.

Bimetallic catalysts combining a strongly hydrogenating metal with a weaker hydrogenating metal have been utilized to increase selectivity compared with the strongly hydrogenating metal catalysts alone. The second metal blocks some of the active sites and can alter the electronic properties of the hydrogenating metal, leading to changes in surface adsorption of the substrate that can be favorable for semihydrogenation through reduction in desorption barriers for the desired intermediate.^62−64^ Huang et al. explored this phenomena by synthesizing PdNi, PdAg, and Pd catalysts on γ-Al_2_O_3_ and β-zeolite (*BEA, pore window 7.6 × 6.6 Å) supports via incipient wetness impregnation.^65^ They found that PdAg catalysts have the highest selectivity on both supports as compared to PdNi and Pd catalysts. Moreover, β-zeolite-supported catalysts gave a higher selectivity to ethylene than their γ-Al_2_O_3_ supported counterparts. Alloying Pd with Ag was suggested to enhance ethylene selectivity by facilitating acetylene adsorption which was supported by the observation of an increase in the equilibrium constant for acetylene hydrogenation over the bimetallic catalyst compared to Pd alone. Additionally, the β-zeolite-supported catalysts showed higher dispersion and smaller particle sizes than the γ-Al_2_O_3_ supported catalysts. Larger metal particles encourage the formation of Pd β-hydride, which promotes overhydrogenation of acetylene to ethane. It was suggested that since the β-zeolite support hosts smaller metal clusters, fewer Pd β-hydride species form, thereby enhancing ethylene selectivity.

In an attempt to replace Pd with an earth abundant metal, Hu et al. created CuNi bimetallic catalysts of various ratios on ZSM-12 (pore window 6.0 × 5.6 Å) support.^66^ First, they found that the combined bimetallic material had enhanced performance compared to its individual constituents. Furthermore, at an optimal temperature of 250 °C with a Ni/Cu ratio of 7, 100% acetylene conversion could be achieved with 82.5% ethylene selectivity as compared to the Ni/ZSM-12 catalyst, which only achieved ∼70% selectivity at full acetylene conversion. Since Cu is a much weaker hydrogenation metal, its activity was much lower than Ni and the bimetallic catalysts. This was made apparent through the observation that Cu could not achieve full acetylene conversion even at temperatures 100 °C greater than what was used for Ni and the bimetallic catalysts at full acetylene conversion. However, its selectivity was greater than that of Ni alone at 78.5%. The bimetallic catalyst achieving full conversion while enhancing selectivity is a prime example of how bimetallic catalysts can promote selective hydrogenations using more earth abundant materials.

Overall, postsynthetic synthesis techniques offer a straightforward method to obtain zeolite confined catalysts at the cost of limited control over the location of TMIs and the formation of metal clusters. Specifically, postsynthetic methods have been shown to form large metal clusters and metal sites located on the external surface of the zeolite rather than within confinements, which decrease catalyst selectivity via over hydrogenation and/or oligomer formation.^5,67^ As seen from the above examples, altering support acidity via varying the Si/Al ratio or by back exchange with alkali metal ions (e.g., Na^+^, K^+^) and utilizing bimetallic catalysts in place of monometallic catalysts can increase ethylene selectivity, but synthesis methods that provide more control of the metal location and speciation are highly desirable.

### 3.2 Hydrothermal Synthesis Techniques

**Figure 4:**
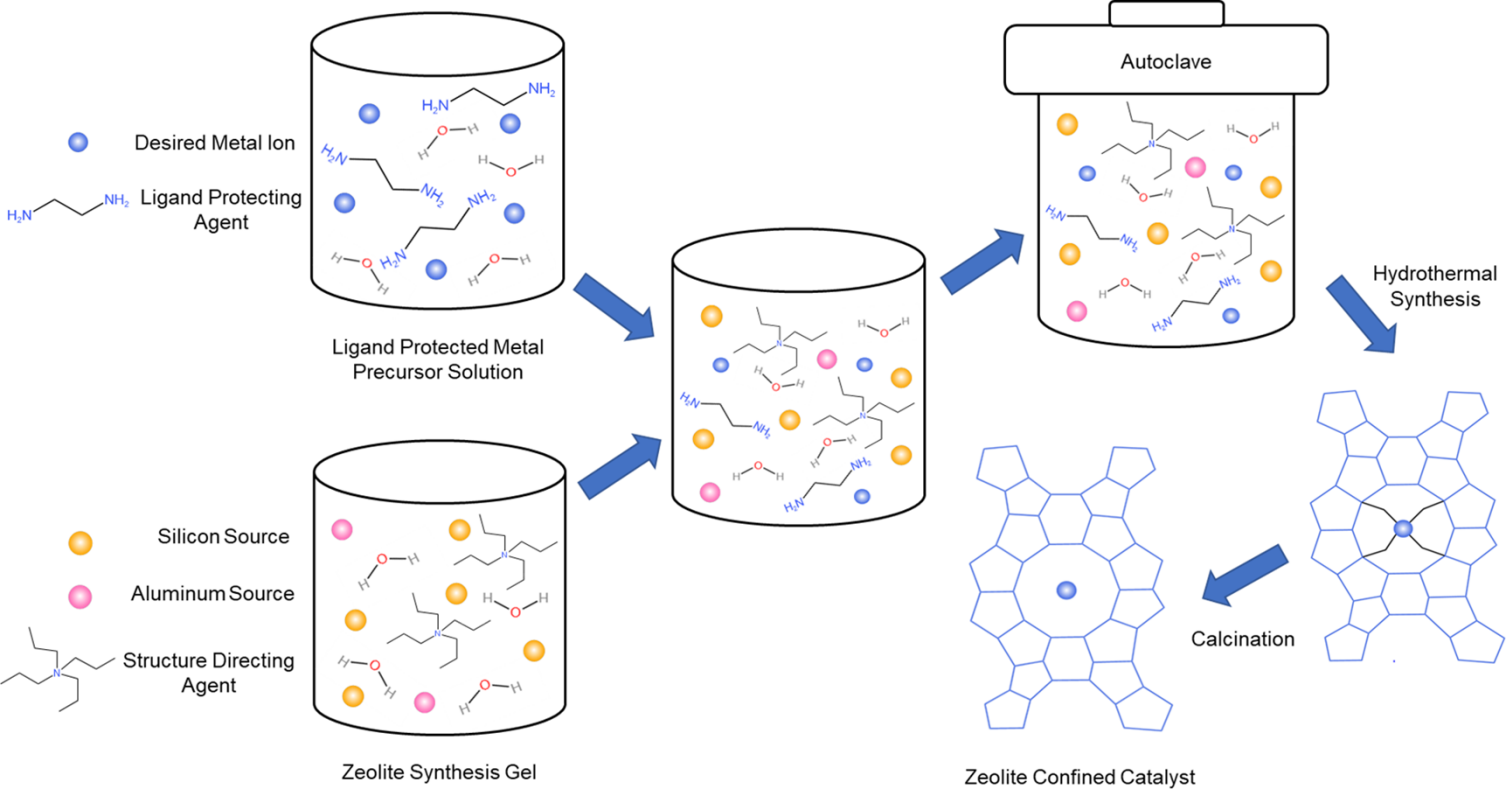
Schematic of hydrothermal synthesis technique for the synthesis of zeolite confined catalysts. The method begins with the preparation of the synthesis gel containing a silica source (e.g., TEOS), and alumina source (e.g., NaAlO_2_) and structure directing agent (e.g., TPAOH). The ligand protected TMI solution is then prepared and added to the synthesis gel. The gel is then transferred to an autoclave to crystallize. Finally, the zeolite is collected and calcined in air to remove the ligands resulting in the zeolite confined catalyst.

Hydrothermal synthesis techniques involve the utilization of structure directing agents (SDAs), such as tetra-propylammonium (TPA), along with SiO_2_, an Al source such as sodium aluminate (NaAlO_2_), and water in a synthesis gel to be crystallized.^36,68−70^ For *in situ* introduction of metal atoms or clusters into zeolite confinements, ligand-protected active metal precursors must be added to the synthesis gel to prevent the precipitation of metal species out of solution.^36^ Amine based molecules, such as diethylenetriamine (DETA) or ethylenediamine (EDA), are often used to stabilize the metal cations because of their stability in the high pH solutions of the synthesis gel, ability to interact with the zeolite framework during crystallization, and the ease of removal with calcination under air.^36,50^ Briefly, as shown in Figure 4, a solution of SDA, Al, and SiO_2_ sources is made with the desired Si/Al ratio. A solution of metal precursor such as Ni or Pd nitrates, same as those used for post-synthetic techniques, and a selected ligand protecting agent is made and added to the synthesis gel. The gel is then crystallized in an autoclave typically around 353–443 K for several days and then finally calcined in air at 723–823 K for several hours to remove the amine.^36,71,72^ The location of the metal within the zeolite is regulated by the SDA, ligand, and Si/Al ratio since two Al sites are required to stabilize bivalent metal cations.^72,73^ The location of Al (i.e., Al siting) within the zeolite framework can be difficult to control, so the number of locations suitable to support SACs is often minimal resulting in low transition metal loadings.^55,56^ Recently, Khivantsev and co-workers have shown that the choice of base utilized during synthesis can impact the number of Al pairs found in the framework. Specifically, utilizing Sr(OH)_2_ as opposed to NaOH during the hydrothermal synthesis of SSZ-13 resulted in 55% more Al pairs and increased catalyst stability.^74^

Single atoms catalysts combined with zeolite confinements have shown a lot of promise as active and selective hydrogenation catalysts. For example, Chai et al. synthesized catalysts with single cationic Ni sites inside CHA cages (3.8 × 3.8 Å).^50^ Under optimal conditions of 453 K, gas-hourly space velocity (GHSV) 15 000 h^–1^, and excess hydrogen in a pure acetylene feed, this catalyst was able to achieve 100% acetylene conversion with an astonishing 97% ethylene selectivity. It is worth noting that these conditions included a temperature that is approximately 100 K greater than what is required to achieve near full conversion for the standard catalysts, Pd/Al_2_O_3_ (363 K) and the Lindlar catalyst (333 K), likely due to Ni being a less active metal for this reaction than Pd since all other reaction conditions (e.g., GHSV, catalyst loading) were stated to be constant across catalysts. Similarly, in another work by this group, cationic Ni was confined in a large pore zeolite, Faujasite (FAU pore window 7.4 × 7.4 Å), and subjected to reaction under similar conditions.^45^ It was also able to achieve 100% acetylene conversion, but with a slightly lower selectivity–approximately 92%. The authors noted an apparent difference in the mechanism between the two catalysts. The small pore CHA (pore window 3.8 × 3.8 Å) was suggested to follow the traditional Horiuti-Polanyi mechanism while the larger pore FAU (pore window 7.4 × 7.4 Å) appeared to follow an associative mechanism which is more typical of homogeneous catalysts (*vide infra*). Temperature-programmed desorption (TPD) and Fourier Transform Infrared Spectroscopy (FTIR) studies both confirmed that hydrogen preferentially adsorbs over hydrocarbons on the Ni@CHA catalyst at reaction temperature (463 K), indicating that hydrogen adsorption and subsequent activation are the first steps in the mechanism. Hydrogen–deuterium (H-D) exchange experiments and DFT calculations performed on Ni@CHA and Ni@FAU both showed that Ni@CHA could activate dihydrogen on its own, while Ni@FAU required acetylene to be cofed to see evidence of H-D exchange. Specifically, the Ni@FAU catalyst had a significantly larger energy barrier (112 kJ/mol) when dihydrogen activation was the first step in the mechanism compared to the acetylene-assisted pathway (84 kJ/mol). This fascinating difference in the mechanism was attributed to the smaller pores having a stronger local electric field, thereby allowing for the direct dissociation and activation of dihydrogen molecules. Recently, Lercher and co-workers and Surendranath and co-workers have attempted to develop a framework to elucidate and enumerate the effect of electric fields on reaction kinetics.^75−81^ Specifically, Lercher and co-workers have suggested that the influence of electric fields center around the modulation of ground and transition states inside the confined environments of zeolites.^75−78^ The influence of electric field on ground and transition state energies can be enumerated as the product of the relative difference in the dipole moments of the transition state and the ground state and the local electric field, in line with recent work by Shetty et al., and Deshlahra et al.^82,83^ Surendranath and co-workers probed the spontaneous local electric field at the solid–liquid interfaces of a Pt catalyst through vibrational Stark effect and the open circuit potential (OCP) established as an electric double layer is formed at the metal-liquid interface.^79^ In addition, the OCP is correlated to the metal work-function and the ionic strength in the double layer suggesting that they may be important indicators in probing electric field effects inside the confined environment of the zeolites.^76^

Rather than trapping single atoms in zeolite pores, other works have encapsulated metal nanoparticles within the zeolite structure. Wang et al. encapsulated subnanometer (nm) Pd clusters inside small pore sodalite (SOD, pore window 2.8 × 2.8 Å) zeolite via a hydrothermal route (Pd@SOD).^84^ The catalysts achieved 94.5% selectivity to ethylene at 100% acetylene conversion, which was significantly greater than the 21.5% selectivity achieved by catalysts prepared via the wetness impregnation method (Pd/SOD), suggesting that the hydrothermal route was critical for encapsulating metal clusters inside zeolite pores which was essential for selectivity. Importantly, the pore windows of the SOD are only 2.8 Å in diameter, which will not allow acetylene or ethylene to diffuse through. These authors implied that H_2_ is activated by confined Pd nanoparticles and then transferred to the zeolite surface to hydrogenate the adsorbed alkyne species. DFT calculations revealed easier desorption of ethylene from the zeolite surface compared to that of unconfined Pd along with a higher hydrogenation barrier to ethane for Pd@SOD, leading to a high ethylene selectivity.

Larger bimetallic particles have also been utilized with zeolite supports, but due to their large size, these NPs break the zeolite framework and therefore are not fully encapsulated like single atoms or smaller nanoclusters (Figure 3g). For example, Luo et al. used ethylenediamine to stabilize palladium and copper species during the crystallization of siliceous MFI zeolite, silicalite, (S-1 pore windows 5.1 × 5.5 Å).^85^ Compared to samples produced by an ion exchange method, the trapped nanoparticles had higher selectivity at nearly full acetylene conversion (92.9% compared to 76.3%) but lower activity due to the partial blockage or reduced accessibility of active sites by the zeolite structure. The lower activity is apparent when comparing the temperature required to reach full acetylene conversion across the catalysts at the same metal loading. While PdCu/S-1 and Pd@S-1 achieved full conversion at 125 and 75 °C, respectively, PdCu@S-1 did not reach full conversion until 175 °C. While Pd alone was able to achieve full conversion at a much lower temperature than the alloyed catalysts, the benefit of Cu addition was obvious when considering its selectivity was the lowest of all the catalysts at a mere 52.7%.

The above examples clearly highlight the ability of zeolite confined catalysts to enhance selectivity for ethylene in acetylene semihydrogenation. However, these catalysts are often noted to be less active/have lower reaction rates based on them requiring higher operating temperatures to achieve the same level of conversion that unconfined catalysts can. This can be misleading, at least in part, because confined catalysts, specifically with SACs, are plagued with low loadings due to limited availability of suitable host sites rooted in the requirement of paired Al sites for stability. Often times within the literature, turnover frequency (TOF) or specific rates (reaction rate per gram of catalyst) are not reported, which overlooks what can be a drastic difference in the availability of catalytic species in the reactor. Even despite this discrepancy, the selectivity enhancement seen across the board with zeolite confined catalysts makes them promising candidate materials for highly selective catalysts. Table 1 highlights this by providing a summary of zeolite confined catalyst for the semihydrogenation of acetylene which includes catalyst synthesis technique, conversion, selectivity, and metal loading (additional information on the reaction conditions can be found in Table S1 and zeolite dimensions can be found in Table S2). Notably, the catalysts synthesized via techniques that ensure TMIs location is within the confinements (e.g., ligand supported hydrothermal synthesis) have higher ethylene selectivity regardless of catalyst type (SAC, bimetallic, or nanoparticle) and zeolite support. This suggests that zeolite confinement is responsible for the observed selectivity enhancement, but the underlying molecular interactions between the support and the catalyst remain elusive. Understanding these interactions and their relationship to catalyst structure will help in the design of zeolite confined catalysts for widespread applications.

**Table 1:** Summary of Zeolite Confined Catalysts and their Performance for the Semihydrogenation of Acetylene

Catalyst	Synthesis method	*X*_C2H2_	*S*_C2H4_	Temp (°C)	Catalyst loading (wt %)	ref
Ni/zeolite A	ion exchange	100	NR	NR	NR	(6)
Pd/ZSM-5	ion exchange	30	60	160	0.04	(5)
Pd/Na mordenite	ion exchange	90	30	80	0.04	(5)
Pd/β-zeolite	wetness impregnation	100	NR	25	1.36	(65)
PdAg/β-zeolite	wetness impregnation	100	NR	25	Pd/Ag 1.36/0.75	(65)
PdNi/β-zeolite	wetness impregnation	100	NR	25	Pd/Ni 1.36/1.38	(65)
CuNi_7_/ZSM-12	wetness impregnation	100	82.5	250	Ni/Cu 1.73/0.27	(66)
Ni/ZSM-12	wetness impregnation	100	70.5	250	2	(66)
Cu/ZSM-12	wetness impregnation	59.6	78.5	250	2	(66)
Na–Ni@CHA	hydrothermal	100	97	180	3.5	(50)
K–Ni@CHA	hydrothermal	48	97	210	3.6	(50)
Ni@CHA	hydrothermal	100	91	190	4.4	(50)
Na–Ni/CHA	wetness impregnation	100	58	110	2.7	(50)
Ni@Y	hydrothermal	100	92	195	4.5	(45)
Ni/Y	wetness impregnation	100	46	195	4.5	(45)
Pd@SOD	hydrothermal	100	94.5	150	0.1	(84)
Pd/SOD	wetness impregnation	100	21.5	150	0.1	(84)
PdCu@S-1	hydrothermal	100	92.9	175	Pd/Cu 0.11/0.77	(85)
PdCu/S-1	wetness impregnation	100	76.3	125	Pd/Cu 0.10/1.51	(85)
Pd@S-1	hydrothermal	100	52.7	75	0.1	(85)

## 4 Characterization

A thorough and careful execution of various characterization techniques is essential to provide insights into catalyst properties and develop relationships between the catalyst structure and observed catalytic behavior. A variety of techniques are often utilized to develop a holistic picture of the physical and electronic properties of developed catalysts. *Operando* studies with various techniques are implemented to study the potentially dynamic behavior of active sites including restructuring under reaction conditions. A few of the commonly employed *in situ* and *ex situ* techniques used to characterize zeolite confined catalysts are outlined below.

### 4.1 Electron Microscopy

**Figure 5:**
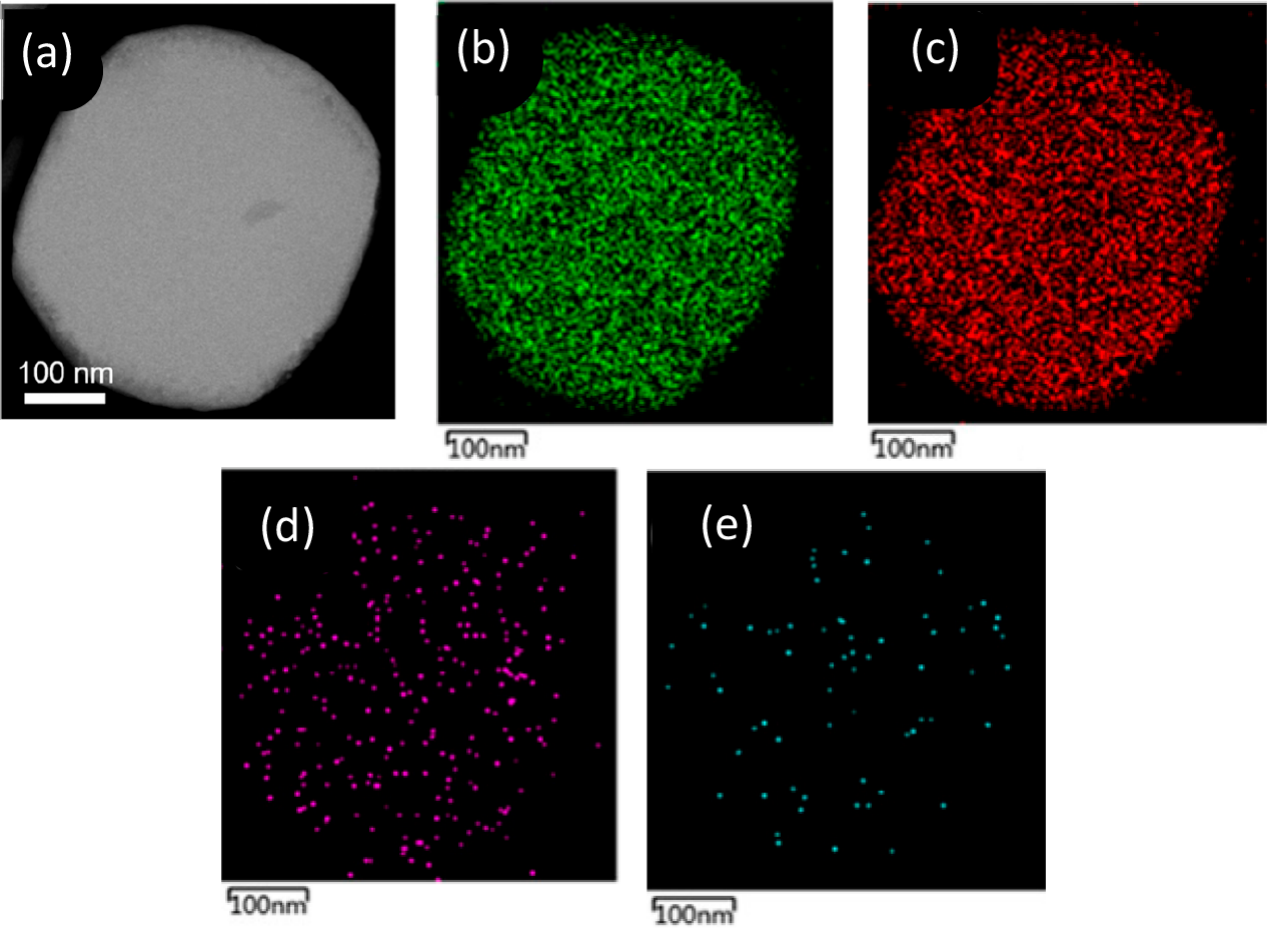
(a) STEM and corresponding element maps of (b) Si, (c) O, (d) Cu, and (e) Pd of PdCu@S-1 catalyst. The element mappings give relative information on location and quantity of various elements in the sample. In this case, one can visualize the low loadings based on the small amount of Cu and Pd in their respective mappings. Additionally, these maps highlight catalyst dispersion on the support. Reproduced/adapted with permission from ref (85). Copyright 2022 American Chemical Society.

Electron microscopy is a useful tool to provide relative local information about the location, dispersion, and loading of active metal on the support. Transmission electron microscopy (TEM) helps in visualizing the location and the nature of transition metal sites in the zeolite framework, but zeolites are not stable under the electron beam during imaging.^36^ Scanning Transmission Electron Microscopy (STEM), which uses lower doses of electrons and shorter acquisition times, thereby keeping the zeolite structure intact, is an attractive alternative technique.^86^ STEM is often paired with elemental mapping such as energy-dispersive X-ray spectroscopy (EDS) (Figure 5). EDS identifies specific elements within a sample based on the unique characteristic X-ray emitted by the element upon excitation with an electron beam.^87,88^ Element mappings provide definitive information on atom location and dispersion within a sample, whereas STEM alone cannot distinguish individual elements especially with catalyst samples that have low loadings or small particles on the support.

**Figure 6:**
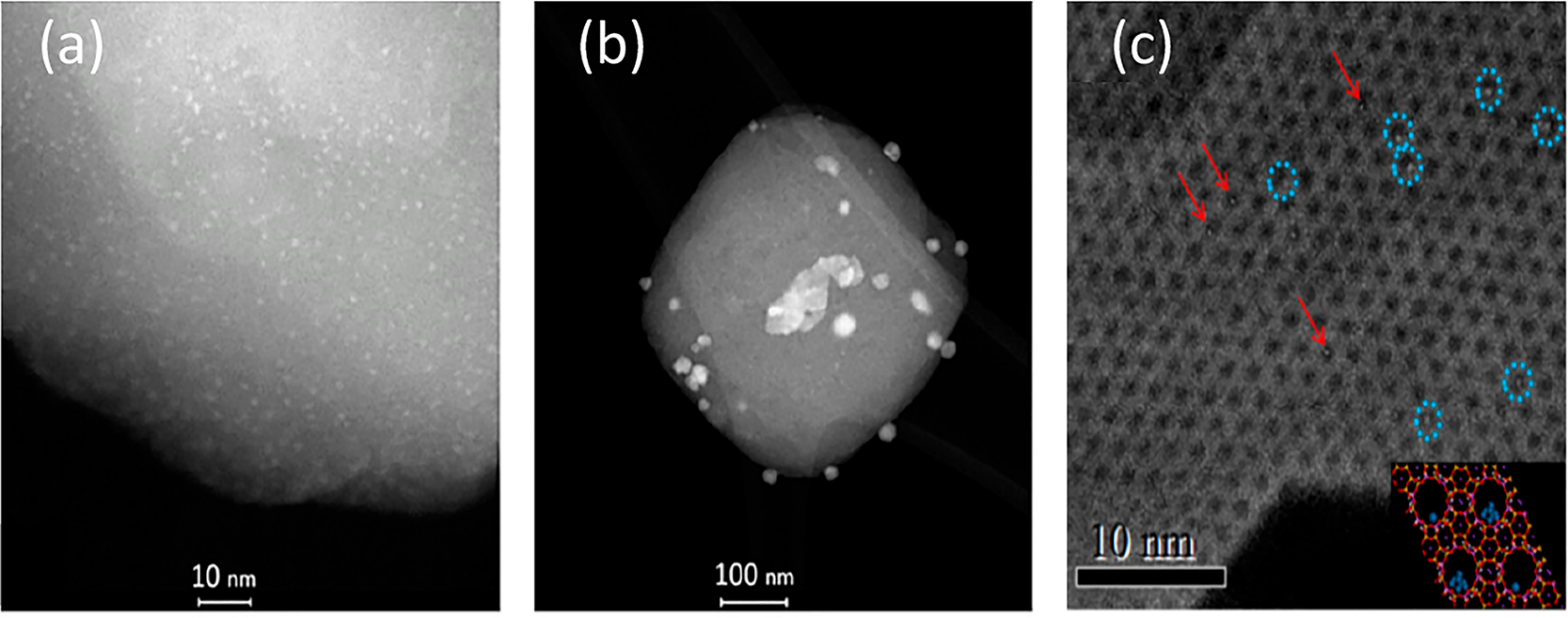
HAADF-STEM images of (a, b) Pt-SSZ-13 and (c) Pt-KL zeolite. Red arrows indicate single Pt atoms, and blue circles Pt clusters. HAADF enhances the Z-contrast, allowing for visualization of transition metal atoms and/or clusters on the zeolite support. (a, b) Reproduced/adapted with permission from ref (89). Copyright 2018 Wiley VCH Verlag GmbH. (c) Reproduced/adapted with permission from ref (91). Copyright 2019 American Chemical Society.

For metals supported on zeolites, using high-angle annular dark field (HAADF) detectors with STEM provides an enhanced Z-contrast compared to TEM (Figure 6).^89^ This provides a brighter signal for the metal on the support when active metals (like Pd, Pt, or Ni) have higher atomic number than the atoms that make up the zeolite support (Si, Al, and O).^87,90^ For example, Xu et al. visualized both Pt atoms and clusters inside the pores of KL zeolite (pore windows 7.1 × 7.1 Å).^91^ As seen in Figure 6c, the Pt sites appeared significantly brighter than the zeolite framework, with Pt atoms (red arrows) and Pt clusters (blue circles) being differentiated after analysis of particle size. To achieve this visualization of atomic species, aberration correctors are implemented to enhance resolution that would otherwise be lost from imperfections in the optical lenses.^88,91,92^ While HAADF-STEM allows for easier visualization of the active sites, visualization of the framework is still lacking. An alternative technique is integrated differential phase-contrast (iDPC) STEM in which the detector detects almost all emitted electrons allowing for simultaneous imaging of high and low atomic number atoms.^93^ Another, more recent development is the use of *in situ* TEM, which allows for microscopy images to be taken under atmosphere.^87,88^ This gives valuable insight into what the active phase of the catalyst may look like when exposed to the reactant gas. However, *in situ* TEM is not a perfect representation of the catalyst under reaction conditions as only near atmospheric pressures and low temperatures can be utilized.^87,88^ Taken together, while these microscopy techniques allow for the visualization of metal species in the zeolite framework, they alone cannot be used to confirm the absence of metal agglomerates since they only provide localized information. As such, they are coupled to other spectroscopic techniques that provide more molecular information.

### 4.2 X-ray Absorption Spectroscopy

X-ray absorption spectroscopy (XAS) is used to provide insights into the coordination environment and valence state of metal sites in zeolite confinements. XAS can reveal the identity of and distance to neighboring atoms from the one of interest (transition metal), which for zeolite frameworks can determine the location of transition metals in the pores.^94,95^ More specifically, the extended X-ray absorption fine structure (EXAFS) gives information on neighboring atoms while X-ray absorption near-edge structure (XANES) provides valence state information.^96^ This along with the ability to analyze samples with less than 1 wt % active metal make it well suited for the study of single atom catalysts on supports.^97^ EXAFS is often used to show a lack of metal–metal (M–M) bonds, thereby confirming the presence of only single atoms on the catalyst support. Additionally, it reveals the coordination of metal sites with the zeolite framework in terms of the number of metal–oxygen (M–O) bonds and their lengths. This information can be utilized in DFT studies to analyze the stability and catalytic activity for potential configurations of metals in zeolite confinements.^96^*In situ* XAS, which shows how oxidation state or location of metal species changes under the reaction environment, can be used to gain insight into what the active phase of the catalyst truly looks like and corroborate DFT studies regarding what probable stable species are under reaction conditions.

While XAS is definitely one of the most useful characterization techniques to investigate SACs on supports, it has its limitations. For instance, the analysis typically does not extend past the first coordination sphere.^97^ As such, it can be near impossible to distinguish the difference between metal–oxygen–metal and metal–oxygen-support bonds.^97^ Many look for the absence of M–M bonds as evidence that only single atom species exist within the catalysts. However, this does not rule out the existence of small metal oxide nanoparticles, as the M–O bonds from framework association would appear almost the same as the M–O bonds present in the metal oxide when analysis does not extend past the first coordination sphere. Additionally, Finzel et al. found that with samples that had both Pt single atoms and nanoclusters, with Pt single atoms being the dominant species, EXAFS was unable to detect the scattering indicative of the Pt–Pt bonds.^97^ This implies that XAS alone may be insufficient to determine the absence of metal oxide and/or metal nanoclusters in the presence of single atoms on support. Overall, XAS provides valuable information about the nature of catalytic species (valence state, SACs vs clusters) and their coordination with the support’s structure. However, since it has been seen to be limited in its ability to definitively determine the absence of metal clusters, pairing with an additional technique that provides specific molecular probing is ideal.

### 4.3 UV–Vis Spectroscopy

UV–vis measures the d-d transition band of the metal ions of interest to give information on their oxidation state and coordination environment.^98^ For zeolite supports, the coordination environment between the metal ion and the lattice oxygen can be probed. Zeolites’ highly ordered nature makes identifying the cages in which TMIs are located possible compared to other supports that are more amorphous. For instance, Schoonheydt et al. has used UV–vis to determine the location of Ni ions within zeolite cages.^99^ They were able to identify the simultaneous presence of tetrahedral- and octahedral-coordinated Ni^+^ in various zeolites. While UV–vis can also be used to identify valence state, it requires that bands for each state must show significant separation to prevent overlap and enable clear identification.^98^ As such, UV–vis alone is often not enough to confirm the valence state of ions but can be used to support this information being obtained from XAS or CO-FTIR.

### 4.4 Fourier Transform Infrared (FTIR) Spectroscopy

**Figure 7:**
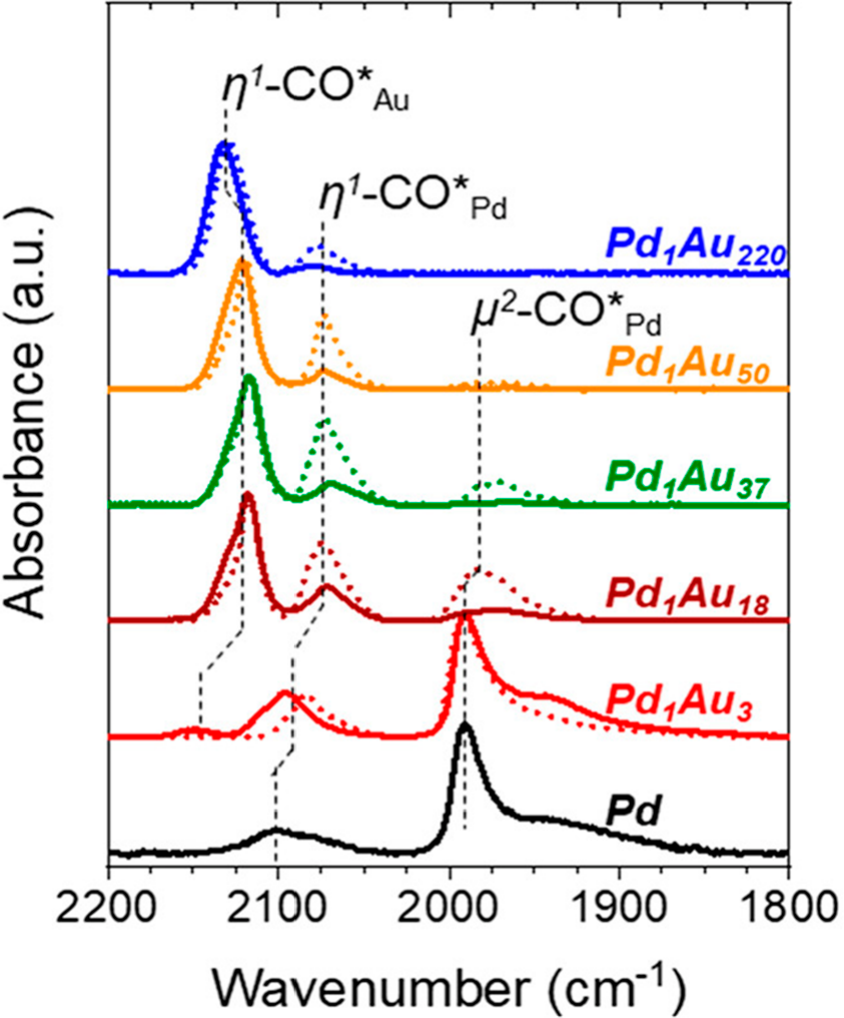
CO-probed FTIR of PdAu alloys with different ratios. The absence of peaks below 2100 cm^–1^ for the Pd_1_Au_220_ alloy suggests the absence of Pd–Pd bonds, which implies the sample only has single atom Pd sites. Reproduced/Adapted with permission from ref (102). Copyright 2021 American Chemical Society.

CO FTIR is used to probe metal speciation and their dispersion on the support.^100^ CO has a unique adsorption wavelength on different metal species thereby allowing for differentiation between single atoms, their different oxidation states, and metal clusters.^26,27^ For example, the different oxidation states of palladium can be distinguished as follows: Pd^2+^–CO: 2215–2140 cm^–1^, Pd^1+^–CO: 2145–2100 cm^–1^, and Pd^0^–CO: below 2100 cm^–1^.^101^ Small Pd clusters are characterized by Pd_*x*_(CO)_*y*_ species with CO linear stretch at around 2000 cm^–1^ and some bridged CO < 1950 cm^–1^.^102^ Absence of stretching frequencies in these regions can be used to confirm the absence of metal clusters as is done in Figure 7, with the lowest Pd/Au.^102^

**Figure 8:**
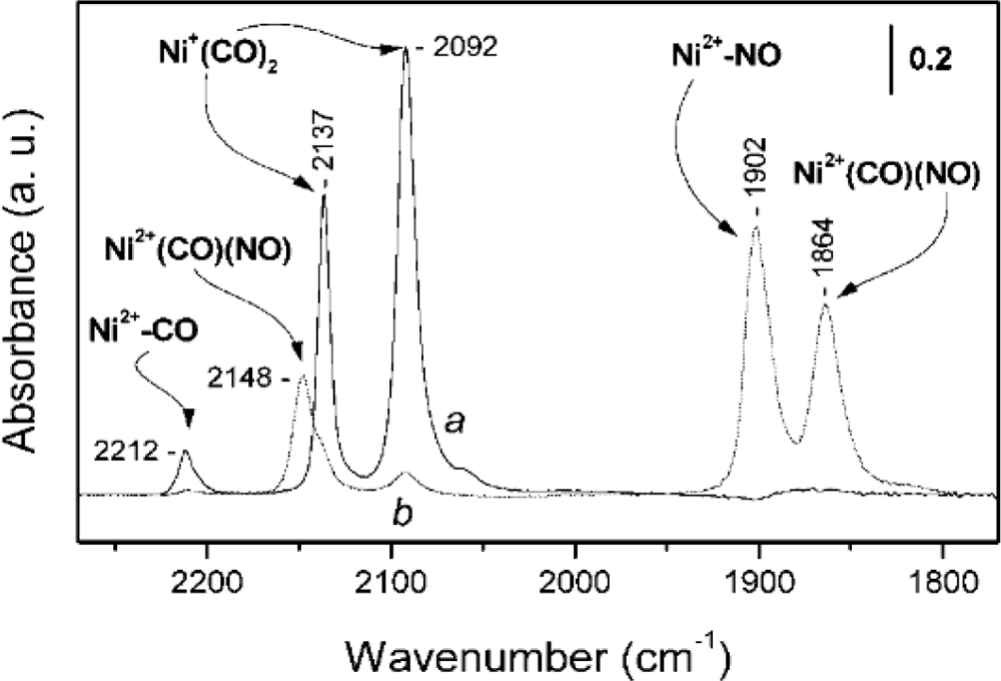
CO and NO probe molecule FTIR of Ni/ZSM-5. Curve (a) is the CO-probed spectra and curve (b) is the same sample following exposure to a small amount of NO after CO adsorption. The reduction in the peak associated with Ni^+^ following NO exposure indicated that the NO molecules oxidize the Ni species. Reproduced/adapted with permission from ref (103). Copyright 2002 American Chemical Society.

This analysis can also be performed with other metals such as Ni, Cu, and Pt.^100^ Ni species confined inside ZSM-5 (5.1 × 5.5 Å) were probed using both CO and NO molecules to determine the Ni oxidation state under different conditions.^103^Figure 8, shows CO adsorption on Ni species before and after exposure to a small dose of NO. Initially, Ni^2+^, identified by a Ni^2+^-CO band at 2212 cm^–1^, and Ni^+^ species, identified by Ni^+^–(CO)_2_ bands at 2137 and 2092 cm^–1^, were both present. Following exposure to NO, several Ni^2+^-NO (1902 cm^–1^) and (NO)(CO) coadsorbed species (2148, 1864 cm^–^1) appeared, but no signal for Ni^+^-(CO)(NO) is observed.^103^ The decrease in the Ni^+^ signal upon NO addition was suggested to indicate NO’s ability to oxidize the metal.

Similar to XAS, performing *in situ* diffuse reflectance infrared Fourier-transform spectroscopy (DRIFTS) can provide insights into the dynamic nature of metal catalysts under reaction conditions.^30^ Chai et al. performed a temperature dependent DRIFTS analysis with their Ni@CHA catalyst for the semihydrogenation of acetylene.^50^ They were able to successfully track the evolution of hydrocarbon species during acetylene hydrogenation, which aligned with the observed relationship between reaction rate and temperature. Specifically, at low temperature (298 K) no evidence of adsorbed hydrocarbon species exist, but upon temperature increases (353–433 K) bands appear at 3010, 2985, and 2965 cm^–1^ which correlate to gas phase ethylene indicating the occurrence of reaction. It should be noted that DRIFTS, like all other techniques discussed, is not free of complexities and limitations. The design and/or selection of the DRIFTS cell must be given careful consideration as the geometry and material can impact the amount of dead volume and temperature gradients within the system, which require calibration/correction of the measured spectra to get accurate results.^104,105^

### 4.5 Co Ion Exchange—Paired Al Quantification

Despite the long-range order and crystalline nature, there is variation in the dispersion and location of Al atoms within the zeolite framework. Al sites can either be isolated—only one Al atom in the framework ring or paired—two Al atoms within the same ring separated by at least one Si atom.^55,57,73^ Rings with paired Al atoms have been suggested by DFT calculations to be the most stable and preferential location for TMIs.^35,36,50^ Quantifying the number of Al pairs in the zeolite framework would provide insight into the potential number of single atom sites that the zeolite support could host. When Co^2+^ is introduced into the zeolite via ion exchange it exclusively titrates paired Al.^73^ Therefore, performing an ion exchange with Co(NO_3_)_2_ and the zeolite sample followed by analysis with inductively coupled plasma (ICP) quantifies the amount of Co^2+^ incorporation and thereby the number of Al pairs.^73,74^ This information offers an upper limit in terms of the potential TMI loading in the form of SACs. Additionally, it can be used to study the impact of synthesis methods and SDAs on the formation of Al pairs in the framework to further tailor the catalyst design to specific loadings of TMIs.

## 5 Probing TMI Behavior *In Situ*

**Figure 9:**
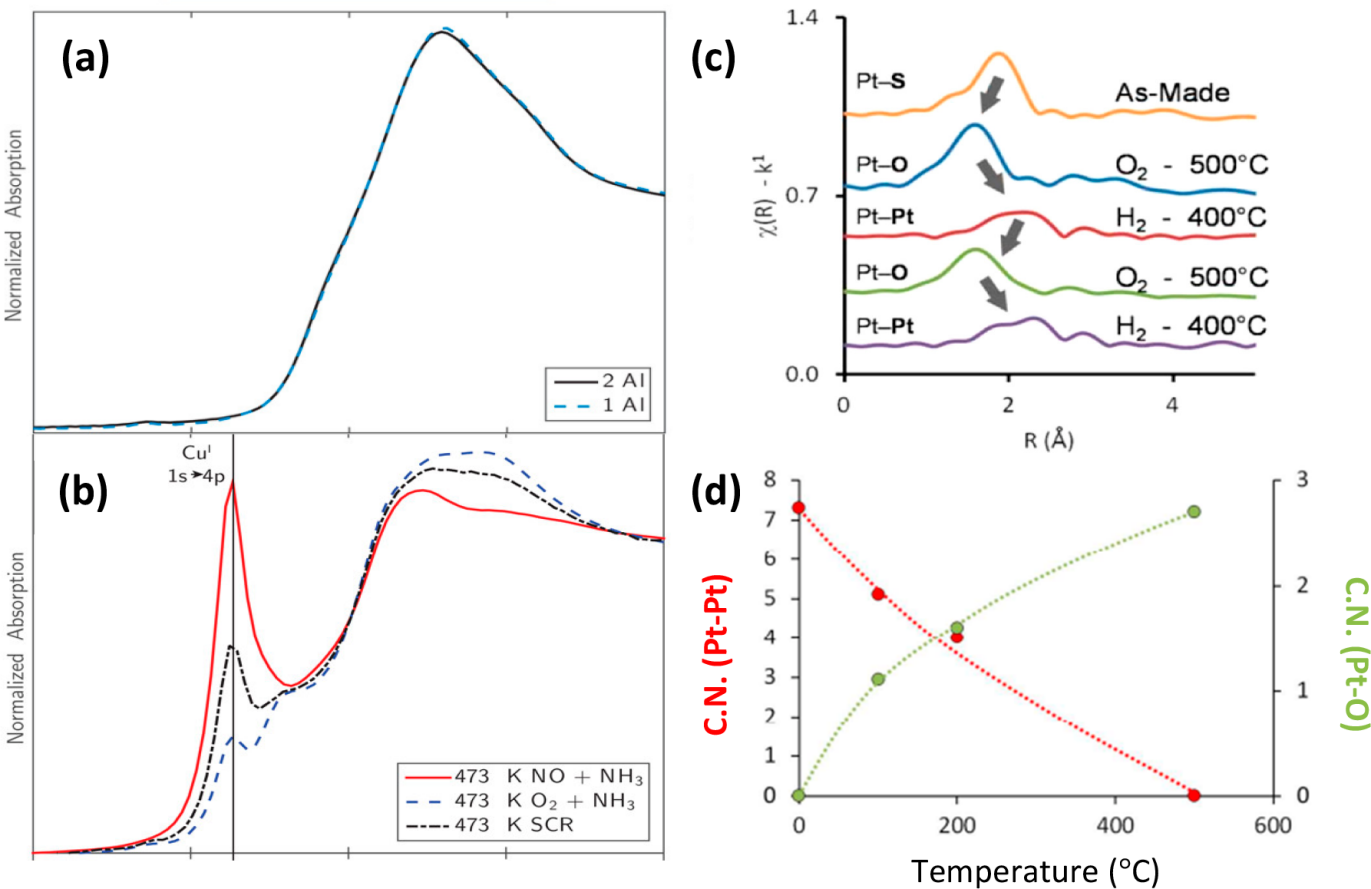
XANES spectra of Cu confined in CHA (Cu@CHA). (a) Cu@CHA under ambient conditions (*ex situ*) and has no indication of Cu(I) and (b) shows Cu coordinated with 1 Al under flow with NO + NH_3_ (red), O_2_ + NH_3_ (blue dashes), and SCR conditions (black dashes). Cu(I) appears in the *operando* spectra despite its absence in the *ex situ* spectra indicating that both Cu(I) and Cu(II) play a catalytic role in the reaction, which is not apparent from the *ex situ* spectra alone. (c) EXAFS of Pt@CHA after various oxidative and reductive treatments showing the formation and disappearance of Pt–O and Pt–Pt bonds. (d) Summary of EXAFS data for Pt@CHA under oxidative conditions as a function of temperature. Labels (c) and (d) were added to the images. (d) was further edited by replacing the axis labels with new text for clarity. (a, b) Reproduced/adapted with permission from ref (107). Copyright 2016 American Chemical Society. (c, d) Reproduced/adapted with permission from ref (108). Copyright 2016 American Chemical Society.

SACs have been known to be unstable under high temperatures and redox conditions leading to migration and agglomeration of metal atoms within the support.^106^ Other studies have reported dynamic behavior of single atom metal species under reaction conditions for zeolite confined Cu and Pt catalysts.^107−109^ For example, Paolucci et al. were able to track the dynamic behavior of Cu ions in SSZ-13 zeolite during the selective catalytic reduction of NO_*x*_ with NH_3_.^107^ Utilizing XANES, they found that under reaction conditions the Cu(II) ions were reduced and their mobility increased compared to analysis performed *ex situ*. *Ex situ* analysis did not detect the presence of any Cu(I) species, but *in situ* analysis showed significant reduction of Cu(II) species to Cu(I) (Figure 9a, b). This implied that Cu(I) also played a catalytic role in the system, which is not apparent from *ex situ* analysis alone. In another example, Moliner et al., showed a reversible transformation between Pt single atoms and clusters under redox conditions.^108^ When exposed to a reductive atmosphere, *operando* XAS showed the formation of Pt–Pt bonds and upon applying oxidative conditions, the signal for Pt–Pt bonds disappeared (Figure 9 c, d). This implies that the active phase for these catalysts if used for a reductive reaction is metal clusters, as opposed to the single atoms identified in *ex situ* analysis. While this dynamic behavior has not been studied for zeolite confined acetylene hydrogenation, based on observations in similar systems, it is plausible that the active site for these materials do not remain as single atoms and the selectivity enhancement is something beyond adsorption mode impacting the thermodynamics of the system. *Operando* spectroscopy can provide insight into the nature of the catalyst under reaction conditions to help elucidate the molecular factors responsible for the selectivity enhancement seen by zeolite confined catalysts. Determination of these specific interactions and structure–function relationships will allow for the informed design of zeolite confined catalysts for a wide variety of reactions.

## 6 Impact of Alkali Metal Ions

As noted in the synthesis section, active sites not located in the desired extra-framework positions can easily cause over hydrogenation of acetylene to ethane. One method to reduce these unconfined surface sites is selective poisoning of the catalyst surface using bulky molecules, but another option is to perform a back exchange with alkali metal ions to replace the weakly bound transition metal cations or remaining protons post synthesis.^110,111^ This has successfully increased selectivity in zeolite confined catalysts synthesized using both post synthetic and hydrothermal synthesis techniques.^14,50,112,113^ The identity of the alkali metal ion has also been observed to have an impact on the activity and selectivity of these catalysts. Huang et al. looked at the impact of exchanging Na^+^ ions with K^+^ ions on their β-zeolite supported catalysts.^113^ They found the K^+^ ions caused an increase in ethylene selectivity with Pd, PdAg, and PdNi catalysts, which they attributed to interactions between the cation and π-bonds that form with substrate adsorption resulting in the adsorption of ethylene becoming less favorable. Contrary to these results, Chai et al. performed a post synthesis ion exchange with Li^+^, Na^+^, and K^+^ and found that the catalyst with the Na^+^ ions showed the highest selectivity at fully acetylene conversion, and that K^+^ ions resulted in a resulted in a relatively poor performance with less than 50% acetylene conversion.^50^ They attributed the reduced activity with K^+^ ion-exchange to its larger size, causing diffusion limitations. While these studies did involve the use of different zeolites that have different structures and pores sizes which could explain some variation in results, they highlight that a clear consensus does not exist on how alkali ions interact with the zeolite support and impact catalyst reactivity and selectivity.

## 7 Liquid Phase Reactions

Similar to reactions in the gas phase, the selectivity of liquid phase reactions can also be increased by taking advantage of the size and shape restrictions imposed by zeolite pores. This has successfully been demonstrated for many molecules such as the preferential hydrogenation of *trans* over *cis* isomers and terminal olefins over internal olefins.^114^ Unique to liquid phase reactions in zeolites compared to gas phase reactions are the interactions that occur between the solvent, substrate and support which can cause additional rate and selectivity enhancement.^115^

For example, Lercher and co-workers studied the dehydration of cyclohexanol using various zeolites and found the rate increased by up to 2 orders of magnitude within the confined environment.^116−118^ They found that the ratio of alcohol to water within the pore was more than 20 times greater than in the bulk environment, which results in favorable conditions for the alcohol dehydration. They also determined that activation enthalpy and entropy increase with increasing pore size, explaining why the highest turnover frequency occurs with the smallest pore zeolite. Additionally, the solvent can interact with the pore/substrate, such that it stabilizes specific transition states or reaction pathways over others. Bregante et al. studied epoxidation reactions within zeolite confinements and found that the reactions that had the highest rates were those that had transition states that disrupted the hydrogen bonding of neighboring water solvent molecules.^119^ This disruption was determined to be entropically favorable compared to those transition states which did not impact hydrogen bonding, thereby resulting in the observed rate and selectivity enhancement.

Continuing to study the unique effects the confined environment has in combination with solvent effects can help the design of selective catalysts in the application of fine chemical synthesis. One such candidate is the selective hydrogenation of 2-methyl-3-butyn-2-ol (MBY) to 2-methyl-3-buten-2-ol (MBE), where MBE is a key component in the synthesis of vitamins A and E, which is typically catalyzed by Pd catalysts and has similar reactivity to acetylene hydrogenation.^120,121^ Solvent effects have been studied in regard to this reaction,^122^ but not within the confines of zeolite pores. Analysis of this reaction under various solvents within zeolite confinements similar to that completed in the above examples could potentially result in selectivity enhancement, as seen by other molecules along with a better understanding of solvent-support interactions.

## 8 Additional Confining Supports

**Figure 10:**
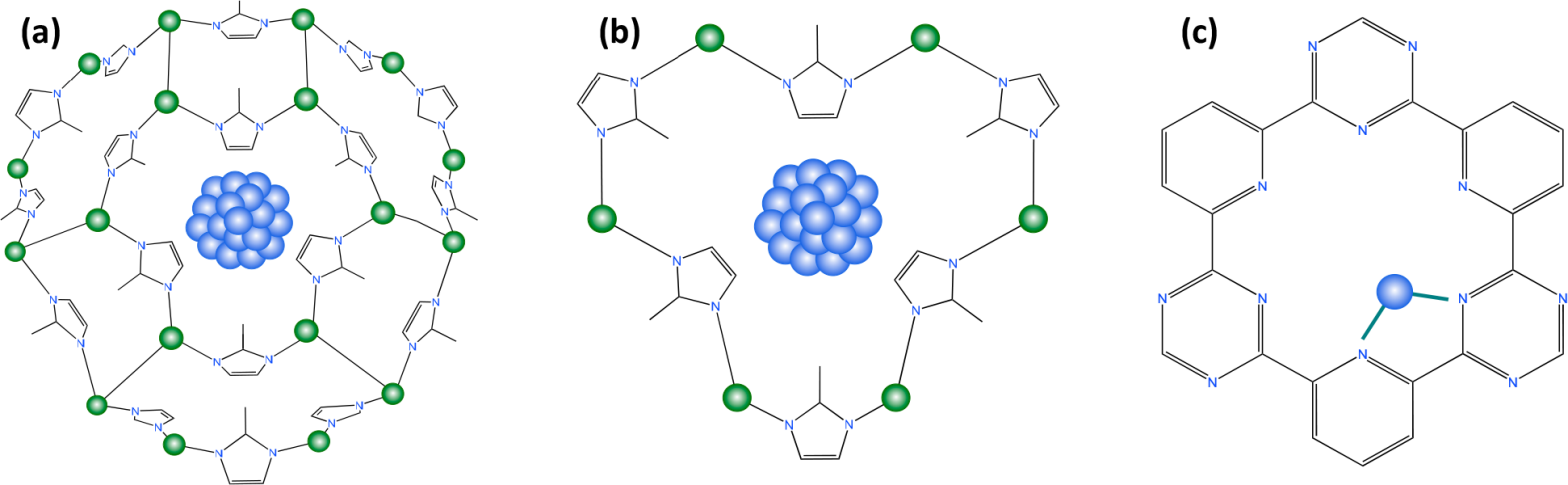
Examples of catalyst confinement in (a, b) MOFs and (c) COFs. (a, b) Metal nanoparticle (blue) confined in the cages of ZIF-8 with green circles representing the framework zinc. (c) Single metal atom confined in the pores of modified CTF. (a, b) Reproduced/adapted with permission from ref (123). Copyright 2021 Elsevier. (c) Reproduced/adapted with permission from ref (129). Copyright 2014 Springer Nature.

Similar to zeolites, metal–organic frameworks, or MOFs, are porous crystalline materials that can be utilized as catalyst supports and offer confinements for stabilization of catalysts (Figure 10 a, b).^123^ MOFs are composed of metal ions or clusters, such as zinc, which are connected by organic ligands like carboxylic acids. The properties of MOFs can be modified by changing the identity of the ligands and/or metal that make up the framework, or through the addition of other species like metal nanoparticles to catalyze a particular reaction.^124−126^ For example, Hu et al.^127^ synthesized PdZn intermetallic nanoparticles of different sizes (1.8–10 nm) and introduced then into ZIF-8C for the semihydrogenation of acetylene. Interestingly, they found that the nanoparticles that were smaller than the size of the MOF pores (<2 nm) did not aggregate at high temperatures (calcination at 600 °C) unlike the larger nanoparticles which showed significant agglomeration post heat exposure. Additionally, these sub 2 nm nanoparticles showed enhanced catalytic activity with greater specific reaction rates along with ethylene selectivity >80%. In a similar study, Liu et al.^128^ confined single Pd atoms within polyoxometalate-based metal–organic framework (POMOF) for the semihydrogenation of acetylene. This catalyst was determined to be highly selective to ethylene, with a selectivity of 92.6% at full acetylene conversion at a temperature of 120 °C. A DFT study on the interaction of molecules and support revealed that the desorption of ethylene had a lower energy barrier than its hydrogenation to ethane thereby allowing for what the authors call a “self-exclusion mechanism”.^128^ This combined with the finding that ethylene preferentially desorbs from the POMOF due to a difference in adsorption energy between acetylene and ethylene was believed to inhibit the over hydrogenation to ethane.

Another class of crystalline porous materials that can be used as catalysts or catalyst supports is covalent–organic frameworks or COFs. COFs are comprised of organic ligands, usually boron, imine, or triazine based, linked together through covalent bonding, but unlike MOFs they do not contain any metal sites.^125^ However, the pores of COFs can still be used to host–metal atoms or nanoparticles creating confined catalysts (Figure 10c).^129^ For example, Zhang et al. used covalent triazine frameworks to confine Pd nanoparticles (<2 nm) for the semihydrogenation of acetylene.^130^ With a mixed feed (1% C_2_H_2_, 20% C_2_H_4_) and under optimized conditions (250 °C, 110 000 h^–1^) the catalyst was able to achieve full acetylene conversion with 83.7% ethylene selectivity. With a pure acetylene feed, over 90% ethylene selectivity was reported. The authors attributed the catalytic performance to a combination of the small Pd nanoparticle size along with the unique effects of confinement and Pd-support interactions. Overall, MOFs and COFs offer easily modifiable support for application in the area of confined catalysis. However, they do tend to be unstable under harsh reaction conditions.^124^ Additionally, analysis regarding the relationship between the confined environment and performance is not well-defined on a molecular level leading to a need to further research into the area to promote molecular understanding of catalyst performance in order to design confined catalysts more intelligently.

## 9 Conclusions

In this review, a variety of catalysts used for the semihydrogenation of acetylene have been summarized with a focus on their preparation method, characterization, and performance. Different active metal sites including single atoms, bimetallic species, nanoclusters, and alloys have all been seen to catalyze the hydrogenation reaction. Additionally, utilizing zeolites as catalyst supports, specifically when catalysts are in extra-framework sites, has been shown to increase the reaction selectivity across various zeolites and metal catalysts while still achieving full acetylene conversion. While transition metal location has been a primary area of focus, other considerations such as the nature of active sites (single atom vs clusters), their potentially dynamic nature, and the identity of charge balancing ions all impact catalyst performance. It is difficult to deconvolute the individual impacts of each of these factors, but with careful and thorough characterization and spectroscopic analysis, major steps can be taken to understand the structure function relationship of these zeolite confined catalysts. Overall, zeolite confined catalysts show the potential to develop both active and selective catalysts because of the unique properties the confinement imposes, and upon deeper understanding of the relationship between confinement and performance, catalysts can be designed more efficiently and for more complex reactions such as those in the liquid phase where additional considerations for solvent interactions are required.

## Supporting Information Available

Tables S1 and S2 referenced in the text with additional information (PDF)
