# Supplementary material for: Challenges and Opportunities for Exploiting the Role of Zeolite Confinements for the Selective Hydrogenation of Acetylene
Source: ACS Appl Mater Interfaces. 2023 Dec 11;16(49):67010–27. doi: 10.1021/acsami.3c11935 (PMC11647899; doi:10.1021/acsami.3c11935)
Supplement: Supplementary file 1 — am3c11935_si_002.pdf [file am3c11935_si_002.pdf]

Supporting Information

Challenges and opportunities for exploiting the role of zeolite confinements for the selective hydrogenation of acetylene

Jenna Vito[1] and Manish Shetty[1*]

[1]Artie McFerrin Department of Chemical Engineering, 100 Spence Street, Texas A&M University College Station, TX 77843, USA.

*Email: manish.shetty@tamu.edu

Number of pages – 9

Number of figures – 0

Number of tables – 2

List of Tables

Table S1. Summary of all catalyst examples discussed in this work including relevant parameters for synthesis, performance, reaction characteristics and reference. (^ = h^{-1}, # = $g_{C2H2}*g_{cat}^{-1}*h^{-1}$, + = s)

Table S2. Summary of dimensions of zeolites discussed in this work. Information from S19 unless otherwise noted.

Table S1. Summary of all catalyst examples discussed in this work including relevant parameters for synthesis, performance, reaction characteristics and reference. (^ = h^{-1}, # = $g_{C2H2}*g_{cat}^{-1}*h^{-1}$, + = s)

Catalyst	Poison (Y/N)	X_{C2H2}	S_{C2H4}	Temperature (K)	Pressure (bar)	GHSV ($mL*g_{cat}^{-1}*h^{-1}$)	Bed loading (g)	metal loading (wt%/M)	C_2H_2 vol%	H_2 vol%	Inert vol%	C_2H_4 vol%	Ref #
Pd/TiO_2	N	98.6	-81	323	1	114000	0.05	1 / Pd	1	25	65	9	S1
PPh3 2.5 Pd/TiO_2	Y	99.9	-5	323	1	114000	0.05	1 / Pd	1	25	65	9	S1
Pd/TiO_2	N	98.6	-81	323	1	114000	0.05	1 / Pd	1	25	65	9	S2
Ph2S Pd/TiO_2	Y	99.4	3	323	1	114000	0.05	1 / Pd	1	25	65	9	S2
Ni/SiO_2	Y	50	55	353	NR	30000	0.2	2 / Ni	2	20	78	0	S3
Ni/SiO_2	N	40	45	453	1	36,000	0.5	8 / Ni	1	5	94	0	S4
PdZn	N	54	88	333	1	180000	0.01	1 / Pd	2	20	38	40	S5
PdZn	N	100	91	373	1	180000	0.01	1 / Pd	2	20	38	40	S5
Pd/Al_2O_3	N	45	12	333	1	1080000	0.005	1 / Pd	2	20	38	40	S5
PdGa	N	86	75	473	1	45000	0.04	100 / PdGa	0.5	5	44.5	50	S6
Pd_2Ga	N	93	76	473	1	45000	0.04	100 / Pd2Ga	0.5	5	44.5	50	S6

Pd_3Ga_7	N	99	71	473	1	45000	0.04	100 / Pd3Ga7	0.5	5	44.5	50	S6
Pd/Al_2O_3	N	43	17	473	1	45000	0.015	5 / Pd	0.5	5	44.5	50	S6
$NiGa/SiO_2$	N	100	82	453	1	15000	0.2	3 / Ni	2	20	78	65	S7
$NiFeCuGaGe/SiO_2$	N	100	98	473	1	30000	0.1	1 / Ni	2	20	78	0	S7
Ni/SiO_2	N	94	55	453	1	36000	0.5	8 / Ni	1	5	94	0	S8
Ni_5Ga/SiO_2	N	100	81	453	1	36000	0.5	8 / Ni5Ga	1	5	94	0	S8
$AgPd0.01/SiO_2$	N	>90	>80	473	1	60000	0.03	0.046 / Pd	1	20	59	20	S9
$AgPd/Al_2O_3$	N	<50	>95	343	1	5400^	0.5	NR	1.46	1.71	15.47	81.36	S9
Pd/PPS	N	100	72	373	1	$0.125^{\#}$	NR	0.3 / Pd	0.9	0.6	49.2	49.3	S10
Pd/SiO_2	N	100	48	373	1	$0.125^{\#}$	NR	0.3 / Pd	0.9	0.6	49.2	49.3	S10
Ni/zeolite A	Y	100	NR	NR	1	$0.5\text{-}1^{+}$	NR	NR	0.35	28	39	34	S11
Pd/ZSM-5	N	30	60	433	1	582	1.5	0.04 / Pd	0.3	0.4	0	99.3	S12
Pd/Na mordenite	N	90	30	353	1	582	1.5	0.04 / Pd	0.3	0.4	0	99.3	S12

Pd/β-zeolite	N	100	NR	298	NR	Batch	0.022	1.36 / Pd	6	35	53	6	S13
Pd-Ag/β-zeolite	N	100	NR	298	NR	Batch	0.022	1.36, 0.75 / Pd,Ag	6	35	53	6	S13
Pd-Ni/β-zeolite	N	100	NR	298	NR	Batch	0.022	1.36,1.38 / Pd,Ni	6	35	53	6	S13
$CuNi_7$/ZSM-12	N	100	82.48	523	NR	9000	0.1	1.73,0.27 / Ni,Cu	33	67	0	0	S14
Ni/ZSM-12	N	100	70.53	523	NR	9000	0.1	2 / Ni	33	67	0	0	S14
Cu/ZSM-12	N	59.59	78.54	523	NR	9000	0.1	2 / Cu	33	67	0	0	S14
Na-Ni@CHA	N	100	97	453	1	15000	0.2	3.5 / Ni	1	16	83	0	S15
K-Ni@CHA	N	48	97	483	1	15000	0.2	3.6 / Ni	1	16	83	0	S15
Ni@CHA	N	100	91	463	1	15000	0.2	4.4 / Ni	1	16	83	0	S15
Na-Ni/CHA	N	100	58	383	1	15000	0.2	2.7 / Ni	1	16	83	0	S15
Ni@Y	N	100	92	468	1	0.48^+	0.3	4.5 / Ni	1	20	79	0	S16
Ni/Y	N	100	46	468	1	0.48^+	0.3	4.5 / Ni	1	20	79	0	S16
Pd@SOD	N	100	94.5	423	1	30000*	0.3	0.1 / Pd	0.6	6	93.4	0	S17

Pd/SOD	N	100	21.5	423	1	30000*	0.3	0.1 / Pd	0.6	6	93.4	0	S17
PdCu@S-1	N	100	92.9	448	1	40000	0.03	0.11, 0.77 / Pd, Cu	1	10	89	0	S18
PdCu/S-1	N	100	76.3	398	1	40000	0.03	0.10, 1.51 / Pd,Cu	1	10	89	0	S18
Pd@S-1	N	100	52.7	348	1	40000	0.03	0.1 / Pd	1	10	89	0	S18

Table S2. Summary of dimensions of zeolites discussed in this work. Information from S19 unless otherwise noted.

Framework Codes	Channel Dimension/ Pore Size (Å)	Size of Pore ring (X MR)	Zeolite Materials with Framework
SOD	2.8x2.8[S17]	6 MR	Sodalite
CHA	3.8x3.8	6 MR	Chabazite, SSZ-12
LTA	4.1x4.1	6 MR	Zeolite A
MWW	4x5.5, 4.1x5.1	6 MR	MCM-22
MFI	5.1x5.5, 5.3x5.5	10 MR	ZSM-5, Silicalite (S-1)
MTW	6.0x5.6	12 MR	ZSM-12
MOR	5.7x2.6, 7.0x6.5	8, 12 MR	Mordinite
*BEA	7.6x6.4, 5.5x5.5	12 MR	β-Zeolite
LTL	7.1x7.1	12 MR	Zeolite L, KL
FAU	7.4x7.4	12 MR	Faujasite, Zeolite X, Zeolite Y

References

S1. J. McCue, A.; McKenna, F.-M.; A. Anderson, J. Triphenylphosphine: A Ligand for Heterogeneous Catalysis Too? Selectivity Enhancement in Acetylene Hydrogenation over Modified Pd/TiO 2 Catalyst. *Catalysis Science & Technology* **2015**, *5* (4), 2449–2459. https://doi.org/10.1039/C5CY00065C

S2. McKenna, F.-M.; Anderson, J. A. Selectivity Enhancement in Acetylene Hydrogenation over Diphenyl Sulphide-Modified Pd/TiO2 Catalysts. Journal of Catalysis 2011, 281 (2), 231–240. https://doi.org/10.1016/j.jcat.2011.05.003

S3. Trimm, D. L.; Liu, I. O. Y.; Cant, N. W. The Selective Hydrogenation of Acetylene over a Ni/SiO2 Catalyst in the Presence and Absence of Carbon Monoxide. *Applied Catalysis A: General* **2010**, *374* (1), 58–64. https://doi.org/10.1016/j.apcata.2009.11.030.

S4. Chen, Y.; Chen, J. Selective Hydrogenation of Acetylene on SiO2 Supported Ni-In Bimetallic Catalysts: Promotional Effect of In. *Applied Surface Science* **2016**, *387*, 16–27. https://doi.org/10.1016/j.apsusc.2016.06.067.

S5. Zhou, H.; Yang, X.; Li, L.; Liu, X.; Huang, Y.; Pan, X.; Wang, A.; Li, J.; Zhang, T. PdZn Intermetallic Nanostructure with Pd–Zn–Pd Ensembles for Highly Active and Chemoselective Semi-Hydrogenation of Acetylene | ACS Catalysis. *ACS Catal.* **2015**, *6* (2), 1054–1061. https://doi.org/10.1021/acscatal.5b01933.

S6. Armbrüster, M.; Kovnir, K.; Behrens, M.; Teschner, D.; Grin, Y.; Schlögl, R. Pd−Ga Intermetallic Compounds as Highly Selective Semihydrogenation Catalysts. *J. Am. Chem. Soc.* **2010**, *132* (42), 14745–14747. https://doi.org/10.1021/ja106568t.

S7. Ma, J.; Xing, F.; Nakaya, Y.; Shimizu, K.; Furukawa, S. Nickel-Based High-Entropy Intermetallic as a Highly Active and Selective Catalyst for Acetylene Semihydrogenation. *Angewandte Chemie International Edition* **2022**, *61* (27), e202200889. https://doi.org/10.1002/anie.202200889.

S8. Wang, L.; Li, F.; Chen, Y.; Chen, J. Selective Hydrogenation of Acetylene on SiO2-Supported Ni-Ga Alloy and Intermetallic Compound. Journal of Energy Chemistry 2019, 29, 40–49. https://doi.org/10.1016/j.jechem.2018.02.001

S9. Pei, G. X.; Liu, X. Y.; Wang, A.; Lee, A. F.; Isaacs, M. A.; Li, L.; Pan, X.; Yang, X.; Wang, X.; Tai, Z.; Wilson, K.; Zhang, T. Ag Alloyed Pd Single-Atom Catalysts for Efficient Selective Hydrogenation of Acetylene to Ethylene in Excess Ethylene. ACS Catal. 2015, 5 (6), 3717–3725. https://doi.org/10.1021/acscatal.5b00700

S10. Lee, S.; Shin, S.-J.; Baek, H.; Choi, Y.; Hyun, K.; Seo, M.; Kim, K.; Koh, D.-Y.; Kim, H.; Choi, M. Dynamic Metal-Polymer Interaction for the Design of Chemoselective and Long-Lived Hydrogenation Catalysts. Science Advances 2020, 6 (28), eabb7369. https://doi.org/10.1126/sciadv.abb7369.

S11. Corbin, D. R.; Abrams, L.; Bonifaz, C. Designing Zeolite Catalysts for Size- and Shape-Selective Reactions: Selective Hydrogenation of Acetylene in the Presence of Butadiene and Ethylene. *Journal of Catalysis* **1989**, *115* (2), 420–429. https://doi.org/10.1016/0021-9517(89)90046-8.

S12. Denkewicz, R. P.; Weiss, A. H.; Kranich, W. L. Palladium Zeolites as Acetylene Hydrogenation Catalysts. *Journal of the Washington Academy of Sciences* **1984**, *74* (1), 19–26.

S13. Huang, W.; McCormick, J. R.; Lobo, R. F.; Chen, J. G. Selective Hydrogenation of Acetylene in the Presence of Ethylene on Zeolite-Supported Bimetallic Catalysts. *Journal of Catalysis* **2007**, *246* (1), 40–51. https://doi.org/10.1016/j.jcat.2006.11.013.

S14. Hu, S.; Zhang, C.; Wu, M.; Ye, R.; Shi, D.; Li, M.; Zhao, P.; Zhang, R.; Feng, G. Semi-Hydrogenation of Acetylene to Ethylene Catalyzed by Bimetallic CuNi/ZSM-12 Catalysts. *Catalysts* **2022**, *12* (9), 1072. https://doi.org/10.3390/catal12091072

S15. Chai, Y.; Wu, G.; Liu, X.; Ren, Y.; Dai, W.; Wang, C.; Xie, Z.; Guan, N.; Li, L. Acetylene-Selective Hydrogenation Catalyzed by Cationic Nickel Confined in Zeolite. *J. Am. Chem. Soc.* **2019**, *141* (25), 9920–9927. https://doi.org/10.1021/jacs.9b03361.

S16. Deng, X.; Bai, R.; Chai, Y.; Hu, Z.; Guan, N.; Li, L. Homogeneous-like Alkyne Selective Hydrogenation Catalyzed by Cationic Nickel Confined in Zeolite. *CCS Chemistry* **2021**, *4* (3), 949–962. https://doi.org/10.31635/ccschem.021.202100820.

S17. Wang, S.; Zhao, Z.-J.; Chang, X.; Zhao, J.; Tian, H.; Yang, C.; Li, M.; Fu, Q.; Mu, R.; Gong, J. Activation and Spillover of Hydrogen on Sub-1 Nm Palladium Nanoclusters Confined within Sodalite Zeolite for the Semi-Hydrogenation of Alkynes. *Angewandte Chemie International Edition* **2019**, *58* (23), 7668–7672. https://doi.org/10.1002/anie.201903827.

S18. Luo, Q.; Wang, H.; Wang, L.; Xiao, F.-S. Alloyed PdCu Nanoparticles within Siliceous Zeolite Crystals for Catalytic Semihydrogenation. *ACS Mater. Au* **2022**, *2* (3), 313–320. https://doi.org/10.1021/acsmaterialsau.1c00080.

S19. Baerlocher, C.; McCusker, L. B.; Olson, D.; Meier, W. M. *Atlas of Zeolite Framework Types*, 6th rev. ed.; Published on behalf of the Structure Commission of the International Zeolite Association by Elsevier: Amsterdam Boston, 2007.
